# Role of Pentraxin 3 in Shaping Arthritogenic Alphaviral Disease: From Enhanced Viral Replication to Immunomodulation

**DOI:** 10.1371/journal.ppat.1004649

**Published:** 2015-02-19

**Authors:** Suan-Sin Foo, Weiqiang Chen, Adam Taylor, Kuo-Ching Sheng, Xing Yu, Terk-Shin Teng, Patrick C. Reading, Helen Blanchard, Cecilia Garlanda, Alberto Mantovani, Lisa F. P. Ng, Lara J. Herrero, Suresh Mahalingam

**Affiliations:** 1 Institute for Glycomics, Griffith University, Gold Coast, Australia; 2 Singapore Immunology Network, Agency for Science, Technology and Research (A*STAR), Biopolis, Singapore; 3 WHO Collaborating Centre for Reference and Research on Influenza, Peter Doherty Institute for Infection and Immunity, Melbourne, Victoria, Australia; 4 Humanitas Clinical and Research Center, Department of Inflammation and Immunology, Rozzano, Italy; 5 Department of Biotechnology and Translational Medicine, University of Milan, Milano, Italy; 6 Department of Biochemistry, Yong Loo Lin School of Medicine, National University of Singapore, Singapore; NIH, UNITED STATES

## Abstract

The rising prevalence of arthritogenic alphavirus infections, including chikungunya virus (CHIKV) and Ross River virus (RRV), and the lack of antiviral treatments highlight the potential threat of a global alphavirus pandemic. The immune responses underlying alphavirus virulence remain enigmatic. We found that pentraxin 3 (PTX3) was highly expressed in CHIKV and RRV patients during acute disease. Overt expression of PTX3 in CHIKV patients was associated with increased viral load and disease severity. PTX3-deficient (PTX3^-/-^) mice acutely infected with RRV exhibited delayed disease progression and rapid recovery through diminished inflammatory responses and viral replication. Furthermore, binding of the N-terminal domain of PTX3 to RRV facilitated viral entry and replication. Thus, our study demonstrates the pivotal role of PTX3 in shaping alphavirus-triggered immunity and disease and provides new insights into alphavirus pathogenesis.

## Author Summary

Chikungunya virus (CHIKV) and Ross River virus (RRV) are arthropod-borne viruses associated with massive epidemics affecting millions of people worldwide, causing widespread distribution of alphaviral-induced arthritis. The rising prevalence of alphavirus infections and, critically, the lack of therapeutic treatments warrant urgent attention to elucidate the innate immune responses elicited, which serves as the first line of host defense against alphavirus. Ironically, robust innate immune responses have been associated with both protective and pathogenic outcomes. Here, we identified PTX3 as an innate protein involved in acute CHIKV and RRV infection in humans. Using an established acute RRV disease mouse model, we revealed a pathogenic immunoregulatory role of PTX3 which led to enhanced viral infectivity and prolonged disease. Transient overexpression of PTX3 in a human epithelial cell line identified the importance of PTX3 N-terminus in binding RRV and modulating viral entry and replication. Collectively, our study identified a previously undescribed pathogenic role of PTX3 during virus infection and shed insights into the sophisticated innate immune responses launched against virus invasion.

## Introduction

Arthritogenic alphaviruses including Ross River virus (RRV) and chikungunya virus (CHIKV) are the causative agents of the widespread arthropod-borne illnesses, Ross River virus disease (RRVD) and chikungunya fever (CHIKF) respectively [1]. RRV is endemic to Australia, Papua New Guinea and South Pacific islands. An average of ~6,000 cases of RRVD endemic to Australia are reported annually [2], and ~500,000 individuals were infected during its first outbreak in Fiji [3]. CHIKV, which is closely related to RRV, has caused large sporadic outbreaks globally, with the largest recorded outbreak of up to 6.5 million cases in India [4]. Recently, 470,000 suspected and confirmed cases of CHIKF have been reported in the Americas [5]. In both RRVD and CHIKF, clinical symptoms include fever, myalgia, fatigue and maculopapular rash [1,6]. Debilitating persistent polyarthritis is the clinical hallmark of alphaviral diseases, often affecting joints in the hands, wrists, elbows, knees and feet, which can persists for months to years post infection [7–9]. In addition, we have recently identified severe pathological bone loss as another characteristic of alphaviral disease which may contribute to the chronic persistent arthralgia [10]. Emerging clinical evidence has demonstrated an increased tendency of CHIKF patients to develop RA [11], and RRVD patients with pre-existing arthritis such as RA have prolonged rheumatic symptoms after infection [12]. These studies suggested a potential link between alphaviral-induced arthritis and other bone diseases, highlighting alphavirus infection as a possible predisposing risk factor for development of complicated bone disorders [13]. The persistency of debilitating polyarthralgias has a serious impact on quality of life and the economy, with an estimated cost of 34 million euros per year solely in the La Reunion CHIKV outbreak [14]. Symptomatic relief is the only therapeutic option currently available, due partly to a lack of understanding of the immune responses elicited during alphaviral infection.

The cellular and humoral arms of innate immunity serve as the first line of host defense against alphaviral invasion. Despite the importance of the innate immune system in the defense against alphaviral infection, increasing evidence of a pathogenic role for innate mediators has also surfaced over the past few years. Excessive production of soluble innate mediators such as interleukin-6 (IL-6), granulocyte macrophage-colony stimulating factor (GM-CSF), tumor necrosis factor-α (TNF-α), interferon-γ (IFN- γ), macrophage chemoattractant protein-1 (MCP-1) and macrophage migration inhibitory factor (MIF) [15–17] contributes to alphaviral disease pathogenesis. Recent evidence that alphavirus-induced diseases can be exacerbated by overt expression of complement factor 3 (C3) [18] and mannose binding lectins (MBLs) [19] highlights the significance of the complement cascade in modulating alphaviral disease pathogenesis.

Long pentraxin 3 (PTX3) is a pattern recognition molecule which belongs to the humoral arm of innate immunity. PTX3 has a role in all three complement pathways, enhancing the activation, inflammation and cell lysis processes [20]. PTX3 can be secreted by a broad range of cell types including neutrophils [21], monocytes, macrophages and myeloid DCs [22] in response to inflammatory signals such as TNF and IL-1 [23]. Upon pathogen encounter, the release of PTX3 enables cells of monocyte-macrophage lineage to recognize and opsonize the pathogen, presenting it to activated phagocytic cells of the immune system for elimination [24]. Elevated expression of PTX3 has been implicated in many inflammatory and autoimmune diseases, including pulmonary infection [25], giant cell arteritis [26], atherosclerosis [27] and rheumatoid arthritis [28]. Intriguingly, PTX3 is thought to have both protective [29,30] and pathogenic functional roles [31] in the immune system.

PTX3 has a variety of ligands, including complement components, microbial moieties, extracellular matrix proteins, growth factors and P-selectin [16]. The interaction of PTX3 and P-selectin is involved in the regulation of inflammation and leukocyte recruitment through attenuation of polymorphonuclear leukocyte (PML, also known as neutrophils) rolling at sites of inflammation [32]. Consequently, this affects the physiological functions of PMNs in pathogen defense and modulates inflammatory processes.

The role of PTX3 in alphavirus-induced diseases has yet to be established. In this study, we identified the crucial involvement of PTX3 during acute alphaviral infections using specimens from CHIKF and RRVD patients. Characterization of PTX3^-/-^ mice and PTX3-overexpressing HEK 293T cells revealed pathological roles of PTX3 in enhancing viral infectivity during acute RRV infection, which was dependent on the binding interaction between RRV and PTX3. In summary, our data demonstrated the crucial role of PTX3 in modulating alphavirus-induced immune responses and disease manifestation through its N-terminal interaction with the virus particles leading to enhanced viral entry and replication.

## Results

### PTX3 is highly induced in acute CHIKF and RRVD patients

**Fig 1:**
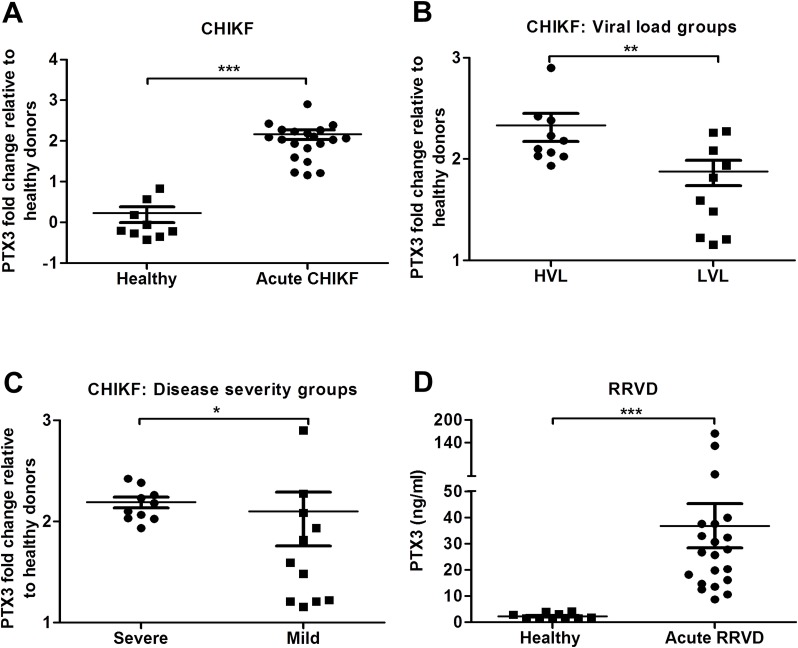
PTX3 expression is elevated in CHIKF and RRVD patients. Expression profile of PTX3 in PBMCs of (A) CHIKF patients (*n* = 20) or healthy controls (*n* = 9) were analyzed by qRT-PCR. Data were normalized to *GAPDH* and shown as fold expression relative to healthy controls. The CHIKF patient cohort was separated into (B) viral load groups: high viral load (HVL; *n* = 10) and low viral load (LVL; *n* = 10), and (C), disease severity group: severe (*n* = 10) vs mild (*n* = 10). (D) Serum from RRVD patients (*n* = 21) or healthy controls (*n* = 10) were analyzed by ELISA for PTX3 levels. Data are presented as mean ± SEM. *P < 0.05, **P < 0.01 and ***P < 0.001, Mann-Whitney *U* test.

Elevated levels of PTX3 have been associated with both protective and pathogenic functions in several inflammatory diseases. To investigate the involvement of PTX3 during acute alphaviral infection, we analyzed PBMCs and serum from CHIKF and RRVD patients for levels of PTX3 using qRT-PCR and ELISA, respectively. Transcriptional expression of PTX3 in PBMCs collected from CHIKF patients was significantly higher compared to controls (Fig. 1A). Further segregation of the CHIKF patient cohort based on viral load (Fig. 1B) and disease severity (Fig. 1C) [15] revealed significantly higher transcriptional expression of PTX3 in patients with higher viral load and more severe disease. Similarly, ELISA analysis of serum specimens collected from acute RRVD patients revealed significantly higher levels of serum PTX3 compared to healthy controls (Fig. 1D). Taken together, these data indicate that PTX3 is induced as part of the innate immune response during acute alphaviral infection and its expression is associated with viral load and disease severity.

### PTX3 is highly induced in an acute RRVD mouse model

**Fig 2:**
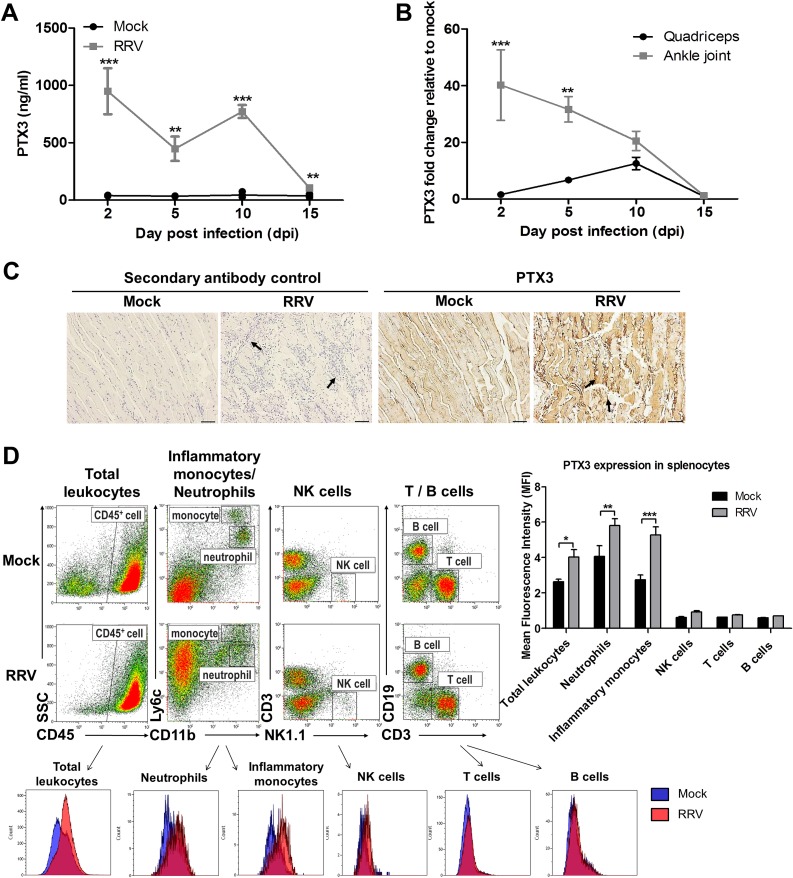
PTX3 expression is up-regulated following RRV infection in murine model. (A) 21-day-old C57BL/6 WT mice (*n* = 4–5 per group) were subcutaneously injected with 10^4^ PFU of RRV or PBS (mock). Mice were sacrificed at 2, 5, 10 and 15 dpi. Serum, quadriceps and ankle joints were harvested. PTX3 expression in serum of RRV- or mock-infected mice was determined by ELISA. (B) Transcriptional profile of PTX3 in quadriceps and ankle joint harvested from RRV- or mock-infected mice at various time points were determined by qRT-PCR. Data were normalized to *HPRT* and shown as fold expression relative to mock-infected. *P < 0.05, **P < 0.01, ***P < 0.001, two-way ANOVA, Bonferroni post-test. Data are presented as mean ± SEM and are representative of 2 independent experiments. (C) Histology of RRV-induced inflammation in quadriceps of WT mice was analyzed by IHC staining with anti-PTX3 antibody at 10 dpi. Arrows indicate abundance of inflammatory infiltrates. Images were taken at 20× magnification. Scale bar, 40 μm. (D) 21-day-old C57BL/6 WT (*n* = 2–3 per group) mice were infected subcutaneously with 10^4^ PFU RRV. Spleens were harvested at 2 dpi and were characterized and quantified by flow cytometry using the markers as described in Materials and Methods to determine mean fluorescence intensity (MFI) of PTX3 expression in total leukocytes, inflammatory monocytes, neutrophils, NK cells, T cells and B cells. Data are presented as mean ± SEM. **P < 0.01, ***P < 0.001, two-way ANOVA, Bonferroni post-test.

To determine the expression of PTX3 during alphaviral disease progression, we utilized an established mouse model of acute RRVD [33]. RRV-infected and mock-infected mice were sacrificed at 2 (peak viremia phase), 5 (disease onset phase), 10 (peak disease phase) and 15 (recovery phase) days post infection (dpi). The serum, quadricep muscles and ankle joints were harvested for analysis. High levels of serum PTX3 were detected in RRV-infected mice across all time points, particularly at 2 and 10 dpi, in contrast to consistently low levels of PTX3 in serum from mock-infected mice (Fig. 2A).

To further investigate PTX3 expression at the sites of inflammation, total RNA was extracted from tissues and analyzed by qRT-PCR. A high level of PTX3 expression was observed at 2 dpi in the ankle joint, with levels declining as the disease progressed. In contrast, quadricep muscles showed peak PTX3 expression at 10 dpi, a time that correlated with the peak of disease (Fig. 2B). IHC was also performed in quadriceps harvested from RRV- and mock-infected mice at 10 dpi (Fig. 2C). Pronounced tissue damage was observed in the striated muscle fibers, which was associated with the presence of inflammatory infiltrates. Increased PTX3 expression was observed in the inflammatory infiltrates of quadricep muscles at peak disease (Fig. 2C).

PTX3 is secreted by a vast array of cell types. To identify the source(s) of PTX3 production during acute RRV infection, we harvested splenocytes from mock- and RRV-infected mice at 2 dpi for flow cytometry analysis. Total leukocytes (CD45^+^) demonstrated significant elevation of intracellular PTX3 after RRV infection. Further segregation of the total leukocytes into various cellular subsets revealed PTX3 induction after RRV infection in only 2 subsets of cells—neutrophils (CD11b^+^ Ly6C^int^) and inflammatory monocytes (CD11b^hi^ Ly6C^hi^). No induction of PTX3 was observed in NK cells (NK1.1^+^ CD3^-^), T cells (CD3^+^ CD19^-^) and B cells (CD3^-^ CD19^+^) (Fig. 2D).

### PTX3 promotes RRV replication *in vivo* and modulates RRV disease kinetics

**Fig 3:**
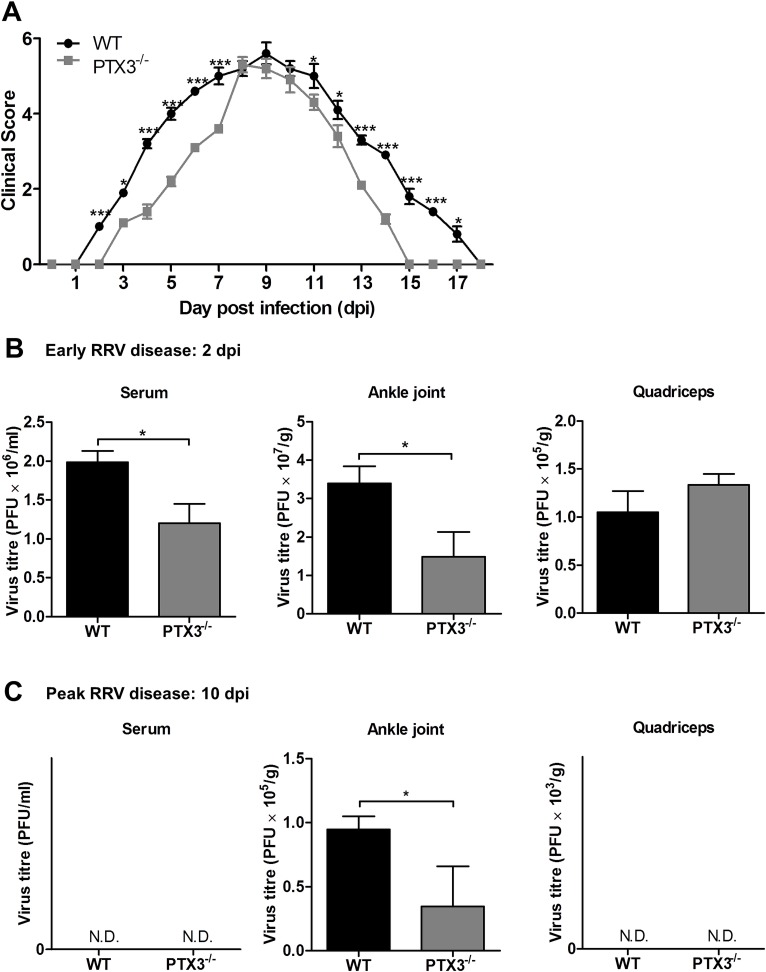
PTX3 modulates RRV replication and disease onset in mice. (A) 21-day-old C57BL/6 WT and PTX3^-/-^ mice were infected subcutaneously with 10^4^ PFU RRV. Disease scores were measured at 24 h intervals. Data are presented as mean ± SEM and are representative of 2 independent experiments. *P < 0.05, **P < 0.01, ***P < 0.001, two-way ANOVA, Bonferroni post-test. RRV titres in serum, ankle joint and quadriceps of RRV-infected WT and PTX3^-/-^ mice (*n* = 3–7 per group) at (B) 2 and (C) 10 dpi were determined by plaque assay. Data are presented as mean ± SEM and are representative of 2 independent experiments. *P < 0.05, **P < 0.01, Student unpaired *t*-test.

High expression of PTX3 during inflammatory diseases has been associated with differential effects [34]. To determine the role of PTX3 in RRV disease, PTX3^-/-^ and wild-type (WT) C57BL/6 mice were infected with 10^4^ PFU RRV and monitored for the development of RRVD clinical signs for up to 18 dpi. Disease onset in RRV-infected WT mice occurred at 3 dpi, with ruffled fur and very mild hind limb weakness (clinical score 2), while in PTX3^-/-^ mice disease onset was significantly delayed commencing at 5 dpi. RRV-infected PTX3^-/-^ mice also demonstrated milder disease signs between 2 to 7 dpi, compared to the RRV-infected WT mice (Fig. 3A). In contrast, there was no significant difference in clinical presentation between PTX3^-/-^ and WT mice during peak disease (from 8 to 10 dpi). From 11 dpi, PTX3^-/-^ mice showed faster disease recovery than WT mice and by 15 dpi regained full function of hindlimbs. In contrast, WT mice continued to display signs of hindlimb weakness until 18 dpi.

To examine the role of PTX3 in modulating RRV replication *in vivo*, viral titre was determined in serum, ankle joints and quadricep muscles harvested at 2 and 10 dpi. As seen in Fig. 3B, viral titres in the serum and ankle joints of RRV-infected PTX3^-/-^ mice were significantly reduced compared to WT mice at 2 dpi. There were no significant differences between PTX3^-/-^ and WT mice in viral titres recovered from the quadricep muscles. At 10 dpi, viral titres recovered from the ankle joints of RRV-infected PTX3^-/-^ mice were also lower than in WT mice. Titres in serum and quadricep muscles from both PTX3^-/-^ and WT mice were below the level of detection at this time (Fig. 3C). To confirm these observations, viral load quantification in ankle joints and quadricep muscles were performed using qRT-PCR. Consistent with previous results, higher viral load was detected in the ankle joints of WT mice at 2 and 10 dpi (S1A Fig.), whereas no difference in viral load was detected between RRV-infected WT and PTX3^-/-^ mice in the quadricep muscles (S1B Fig.).

Collectively, our data indicate that PTX3 deficiency delays the development of RRV clinical signs in infected mice during early infection and assists in rapid recovery in the latter stages of disease. Additionally, the absence of PTX3 also reduced the level of viremia and viral load in the ankle joints of RRV-infected mice.

### PTX3 modulates the expression of inflammatory mediators *in vivo*

**Fig 4:**
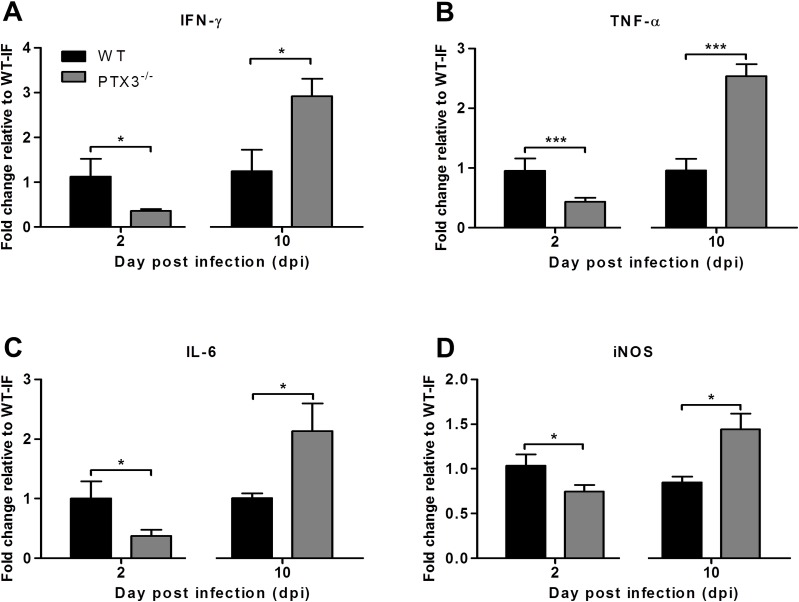
PTX3 modulates expression kinetics of pro-inflammatory mediators during RRV infection in mice. 21-day-old C57BL/6 WT and PTX3^-/-^ (*n* = 4–7 per group) mice were infected subcutaneously with 10^4^ PFU RRV. Transcriptional profiles of immune mediators, (A) IFN-Ɣ, (B) TNF-α, (C) IL-6 and (D) iNOS were determined by qRT-PCR in the quadriceps at early RRV disease (2 dpi) and peak RRV disease (10 dpi). Data were normalized to *HPRT* and shown as fold expression relative to WT. Data are presented as mean ± SEM. *P < 0.05, ***P < 0.001, Student unpaired *t*-test.

We next sought to determine the effects of PTX3 on the expression of inflammatory mediators IFN-Ɣ, TNF-α, IL-6 and iNOS in the early and late phases of RRVD. The quadricep muscles were collected from RRV-infected PTX3^-/-^ and WT mice at early (2 dpi) and peak (10 dpi) RRV disease. At 2 dpi, IFN-Ɣ (Fig. 4A), TNF-α (Fig. 4B), IL-6 (Fig. 4C) and iNOS (Fig. 4D) levels were significantly reduced in RRV-infected PTX3^-/-^ mice. However, at 10 dpi, IFN-Ɣ, TNF-α, IL-6 and iNOS levels were significantly upregulated in RRV-infected PTX3^-/-^ mice compared to WT animals. Collectively, these data demonstrate that the absence of PTX3 results in delayed inflammatory responses in quadricep muscles of RRV-infected mice, as well as enhanced production of these immune mediators in the latter stages of infection.

### PTX3 delays cellular infiltrates recruitment *in vivo*

**Fig 5:**
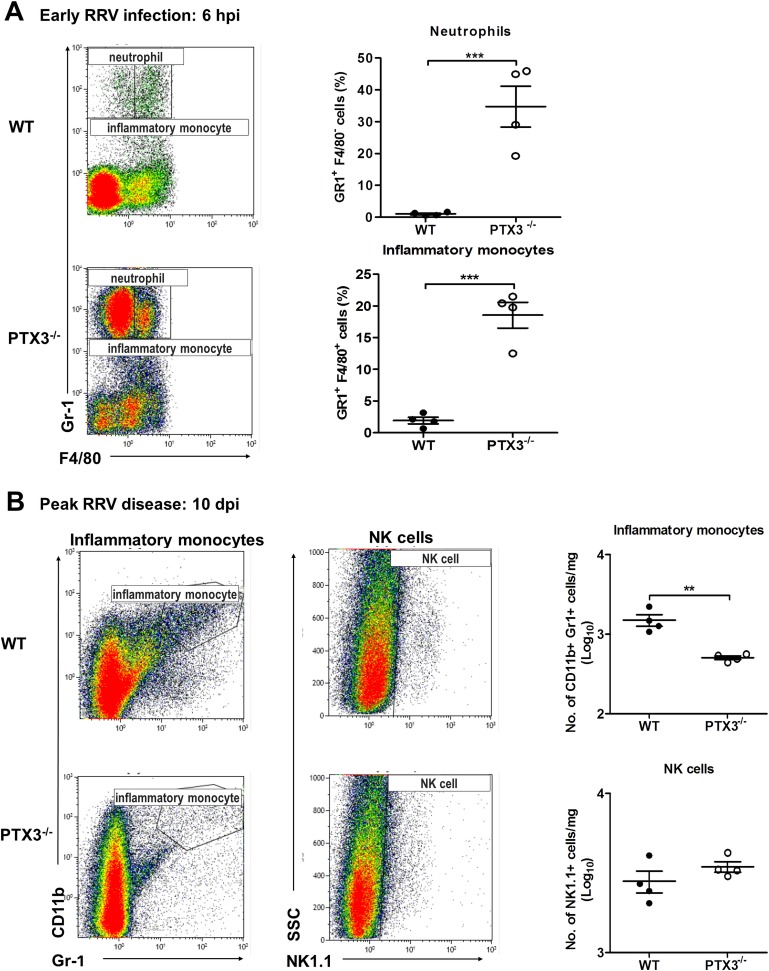
PTX3 delays cellular infiltration kinetics during RRV infection in mice. (A) 6–7 week old C57BL/6 WT and PTX3^-/-^ (*n* = 4 per group) mice were infected intraperitoneally with 10^5^ PFU RRV. Peritoneal lavage harvested at 6 hpi was characterized and quantified by flow cytometry using the markers as described in Materials and Methods to determine percentages of neutrophils and inflammatory monocytes. Data are presented as mean ± SEM. ***P < 0.001, Student unpaired *t*-test. (B) 21-day-old C57BL/6 WT and *PTX3* ^-/-^ (*n* = 4–7 per group) mice were infected subcutaneously with 10^4^ PFU RRV. Leukocytes were isolated from the quadriceps harvested at 10 dpi. Cells were characterized and quantified by flow cytometry using the markers as described in Materials and Methods. Total numbers of inflammatory monocytes and NK cells are shown. Data are presented as mean ± SEM. *P < 0.05 **P < 0.005, Student unpaired *t*-test.

Having demonstrated the effect of PTX3 on the induction of soluble inflammatory mediators during acute RRV infection, we next investigated the effect of PTX3 on leukocyte recruitment during *in vivo* infection. As shown in Fig. 2C, localized cellular infiltration in quadricep muscles of RRV-infected mice occurs at peak disease (10 dpi). To examine the effect of PTX3 on cellular recruitment during early RRV infection, mice were inoculated via the peritoneal route with RRV. At 6 hpi, flow cytometry analysis of peritoneal lavages revealed significantly increased numbers of neutrophils and inflammatory monocytes in the peritoneal cavity of RRV-infected PTX3^-/-^ mice compared to WT mice (Fig. 5A). This early influx of neutrophils and inflammatory monocytes coincides with the chemotactic responses observed in the quadricep muscles of PTX3^-/-^ mice. Among the 5 cytokines investigated, CCL2 and MIF were higher in quadriceps of RRV-infected PTX3^-/-^ mice at 2 dpi compared to WT mice, but not during peak disease (S2A, B Fig.). No significant difference in chemotactic responses of CCL3 (S2C Fig.), CXCL1 (S2D Fig.) and CXCL2 (S2E Fig.) was observed between the PTX3^-/-^ and WT mice at 2 and 10 dpi.

To investigate the effects of PTX3 deficiency on cellular infiltrates during peak RRV disease, mice were infected subcutaneously with 10^4^ PFU RRV and the quadricep muscles examined at 10 dpi. Previously we have shown that inflammatory monocytes and NK cells are the major cells recruited into muscles during localized inflammation [35]. As seen in Fig. 5B, the number of inflammatory monocytes was significantly reduced in PTX3^-/-^ mice compared to WT controls. Infiltration of NK cells, however, was not affected by deficiency of PTX3. Together, these results suggest that acute production of PTX3 dampens early recruitment of neutrophils and inflammatory monocytes, but enhances the egress of inflammatory monocytes in the latter stages of infection.

### PTX3 enhances RRV replication and viral entry *in vitro*

**Fig 6:**
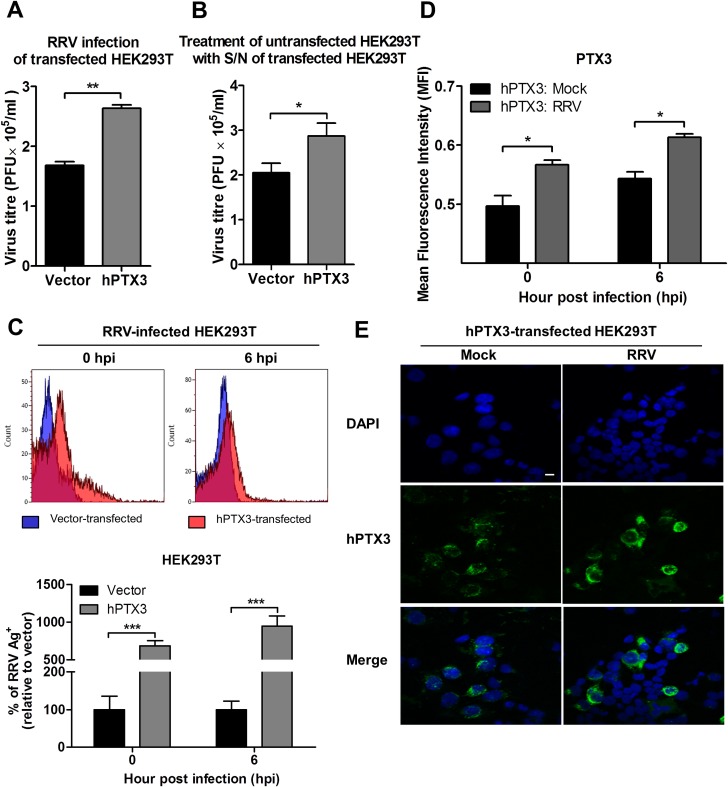
PTX3 enhances RRV replication and viral entry. (A) HEK293T cells were transfected with human PTX3 or vector plasmid for 20 h before RRV infection at MOI 1 for 24 h. Supernatants were harvested and RRV titres determined by plaque assay. (B) Supernatants of transfected HEK293T cells were harvested at 20 h post transfection and used to treat untransfected HEK 293T cells in the presence of RRV (MOI 1) for 1 h at 37°C, followed by 24 h incubation at 37°C in complete medium. Supernatants were harvested and RRV titres determined by plaque assay. Data are presented as mean ± SEM. *P < 0.05 **P < 0.005, Student unpaired *t*-test. Transfected HEK293T cells were harvested at 0 and 6 hpi, (C) quantified by flow cytometry using anti-alphavirus antibody for detection of viral entry, and (D) assessed for intracellular PTX3 expression using flow cytometry analysis. Data (*n* = 3) are presented as mean ± SEM and are representative of 2 independent experiments. ***P < 0.001, two-way ANOVA, Bonferroni post-test. (E) hPTX3-transfected HEK293T cells were fixed at 6 hpi and stained for PTX3 (green) and DAPI. Images are representative of 2 independent experiments. Magnification, ×60. Scale bar, 10 μm.

We next determined the direct effect of PTX3 on the RRV infection process using HEK 293T cells overexpressing PTX3. HEK 293T cells were transiently transfected with a plasmid expressing PTX3 for 20 h and approximately 5 μg/ml of PTX3 could be detected in supernatants using ELISA at this time. In vector-transfected HEK 293T cells, PTX3 could not be detected regardless of RRV infection (S3 Fig.). Overexpression of PTX3 in HEK 293T cells resulted in a significant increase in viral titres recovered from supernatants of RRV-infected cells, compared to cells transfected with control vector, when infected with MOI 0.1, 0.5 and 1 (Fig. 6A, S4A Fig.). This data suggests a direct effect of PTX3 in enhancing RRV replication.

To support that the presence of PTX3 enhanced viral titres, supernatants from vector- and PTX3-overexpressing HEK 293T cells were harvested at 20 h post transfection and incubated with untransfected HEK 293T cells. In the presence of RRV, untransfected HEK 293T cells treated with supernatant from PTX3-overexpressing HEK 293T cells supported significantly increased virus production compared to cells treated with supernatants from vector-treated control cells (Fig. 6B). These data confirmed that the presence of PTX3 is crucial for enhancing virus production.

**Fig 7:**
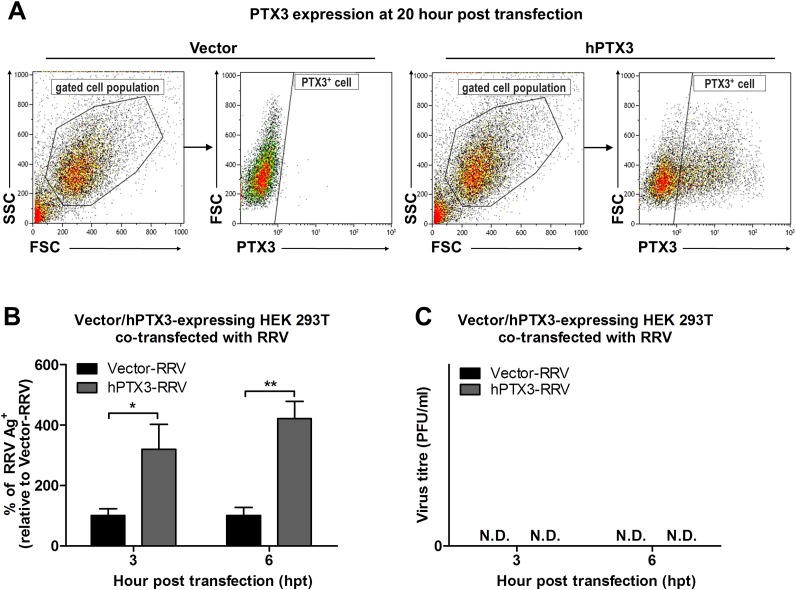
PTX3 promotes early viral replication in PTX3-expressing HEK 293T cells co-transfected with RRV. (A) HEK293T cells were transfected with human PTX3 or vector plasmid for 20 h. Transfected cells were harvested to assess intracellular PTX3 expression using flow cytometry analysis. (B) Vector-/hPTX3-expressing cells were harvested at 20 h post transfection and subjected to a second transfection with RRV through electroporation. Co-transfected cells and supernatant were harvested at 3 and 6 h post RRV transfection and intracellular RRV expression was assessed by flow cytometry using anti-alphavirus antibody. (C) Virus titres in the supernatants were determined by plaque assay. Data (*n* = 3) are presented as mean ± SEM and are representative of 2 independent experiments. ***P < 0.001, two-way ANOVA, Bonferroni post-test.

To confirm that the results of enhanced virus production was due to PTX3 enhancing RRV replication, HEK 293T cells transiently transfected with vector or hPTX3 plasmids were harvested at 20 hour post transfection (hpt) (Fig. 7A) and subjected to a second round of transfection with RRV T48 plasmid through electroporation. At 3 h and 6 h post RRV transfection, cells were harvested for flow cytometry analysis, which demonstrated a significant increase in virus antigen detected within PTX3-, RRV-transfected HEK 293T cells compared to vector-, RRV-transfected control (Fig. 7B). No virus was detected in the supernatant of these RRV-transfected cells at 3 and 6 hpi (Fig. 7C).

To further characterize the effect of PTX3 during alphaviral infection, we examined the potential of PTX3 to directly interact with the virus and enhance viral entry. We quantified the viral load in PTX3-overexpressing HEK 293T cells at early time points following a one-hour virus adsorption step. Typically, alphavirus particles attach to and enter cells during the adsorption phase of infection (0 hpi), with the replication of alphavirus genome commencing 5 to 6 hpi [36]. Therefore, following an hour of virus adsorption, the detection of viral antigens present at 0 hpi is indicative of binding and entry, and 6 hpi is indicative of the synthesis of new virus particles. Detection of intracellular viral antigens in RRV-infected PTX3-overexpressing HEK 293T cells revealed a significant increase in the number of RRV antigen positive cells at 0 and 6 hpi compared to vector-transfected cells (Fig. 6C), indicating that PTX3 facilitates viral entry. This result was further confirmed with qRT-PCR viral load analysis, which detected increased viral load within PTX3-expressing cells at 0, 1, 2, 4, 5 and 6 hpi, compared to vector control (S4B Fig.). At 4 hpi, the first round of virus replication was observed when a sudden spike in viral load was detected (S4B Fig.). Interestingly, in conjunction with increased viral entry in the RRV-infected PTX3-overexpressing cells, we also observed a significant increase in intracellular PTX3 expression, compared to the mocked-infected controls (Fig. 6D, 6E). Furthermore, flow cytometry analysis showed up to 90% of RRV^+^ cells were PTX3^+^, suggesting the co-localization of RRV with PTX3 during acute infection (S5 Fig.). Similar results were obtained for CHIKV infection of PTX3-expressing HEK 293T cells. Enhanced viral titres were recovered from the supernatant of PTX3-expressing CHIKV-infected cells when compared to vector controls (S6A Fig.). Further evaluation of CHIKV-infected cells at 0 and 6 hpi demonstrated significant increase in viral entry in PTX3-expressing cells in conjunction with increased intracellular PTX3 expression (S6B, C Fig.).

**Fig 8:**
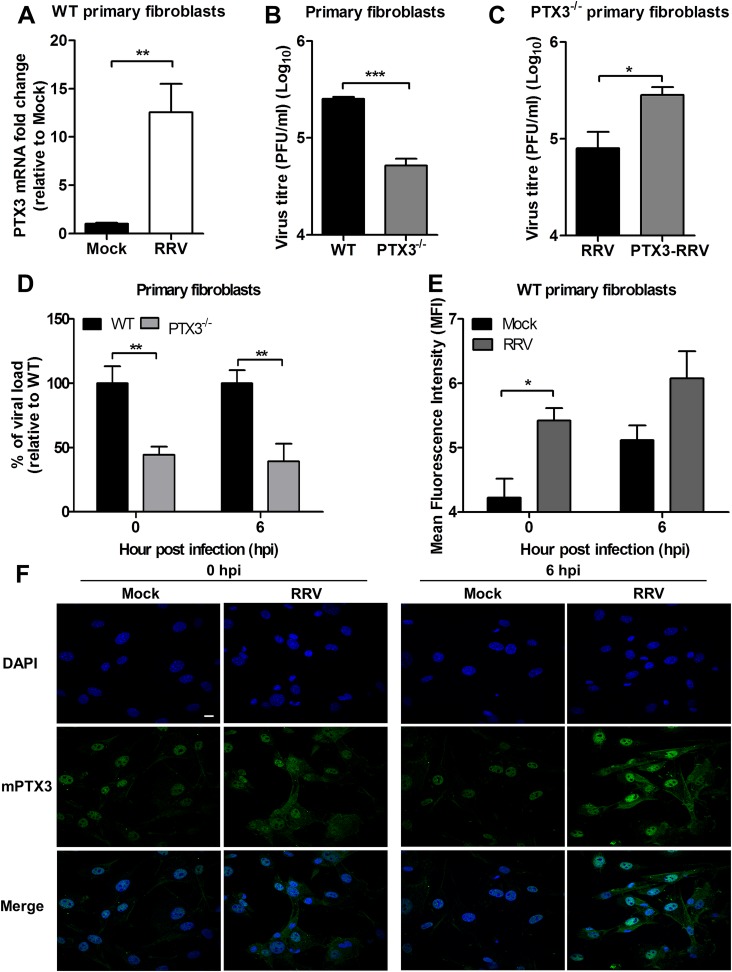
PTX3 enhances RRV replication in murine primary fibroblasts. (A) Primary tail fibroblasts isolated from WT mice were infected with RRV at MOI 1 for 24 hours. Transcriptional profiles of PTX3 in mock- and RRV-infected fibroblasts were determined by qRT-PCR. Data were normalized to *HPRT* and shown as fold expression relative to mock-infected cells. (B) Primary tail fibroblasts isolated from WT and PTX3^-/-^ mice were infected with RRV at MOI 1 for 24 hours. Supernatants were harvested and RRV titres determined by plaque assay. (C) Primary tail fibroblasts from PTX3^-/-^ mice were infected with RRV (10^4^ PFU RRV) and pre-bound PTX3-RRV complex (5 μg/ml of mouse recombinant PTX3 + 10^4^ PFU RRV) for 24 hours. Supernatants were harvested and RRV titres determined by plaque assay. (D) Primary tail fibroblasts from WT and PTX3^-/-^ mice were infected with RRV at MOI 1 and harvested at 0 and 6 hpi for viral load analysis to assess viral entry, using viral load qRT-PCR with specific probe and primers against RRV nsP3 RNA, where total RRV nsP3 copy number was calculated and expressed as a percentage relative to WT infected controls, and (E) assessed for intracellular PTX3 expression using flow cytometry analysis. Data (*n* = 3) are presented as mean ± SEM of percent relative to WT and are representative of 2 independent experiments. *P < 0.05, **P < 0.01, two-way ANOVA, Bonferroni post-test. (F) Primary tail fibroblasts from WT mice were infected with RRV at MOI 1, harvested at 0 and 6 hpi and stained for PTX3 (green) and DAPI (blue). Images are representative of 2 independent experiments. Magnification, ×60. Scale bar, 10 μm.

To demonstrate that the effect of PTX3on enhancing RRV entry and replication contributed to the increased level of virus detected in the *in vivo* studies, we performed RRV infection on primary fibroblasts isolated from tails of PTX3^-/-^ and WT C57BL/6 mice. At 24 hpi, RRV infection of WT fibroblasts resulted in significant up-regulation of PTX3 mRNA expression compared to mock-infected WT fibroblasts (Fig. 8A). Moreover, viral titres in supernatants from WT fibroblasts were significantly enhanced compared to fibroblasts from PTX3^-/-^ mice (Fig. 8B). To further demonstrate the importance of PTX3 in enhancing RRV replication, recombinant mouse PTX3 was pre-incubated with RRV prior to infection of PTX3^-/-^ primary fibroblast cultures. Virus titres recovered from supernatants of PTX3-RRV complex-infected PTX3^-/-^ fibroblasts at 24 hpi were significantly enhanced compared to RRV-infected PTX3^-/-^ fibroblasts (control) (Fig. 8C). Furthermore, the effects of PTX3 deficiency on viral entry into primary fibroblasts during the early stages of infection were examined. Consistent with our earlier findings, significantly lower viral load was detected in PTX3^-/-^ primary fibroblasts compared to WT after RRV infection at both 0 and 6 hpi (Fig. 8D). Similarly, RRV infection of WT fibroblasts led to increased PTX3 expression compared to mock-infected controls at 0 and 6 hpi (Fig. 8E). Immunofluorescence staining of the WT fibroblasts also revealed more intense expression of PTX3, particularly within the cytoplasm, after RRV infection at 0 and 6 hpi (Fig. 8F).

Collectively, these data demonstrate that PTX3 promotes viral entry and replication at the early stages of RRV infection (0 and 6 hpi) within host cells.

### PTX3 binds and colocalizes with RRV in the cytoplasm during infection

**Fig 9:**
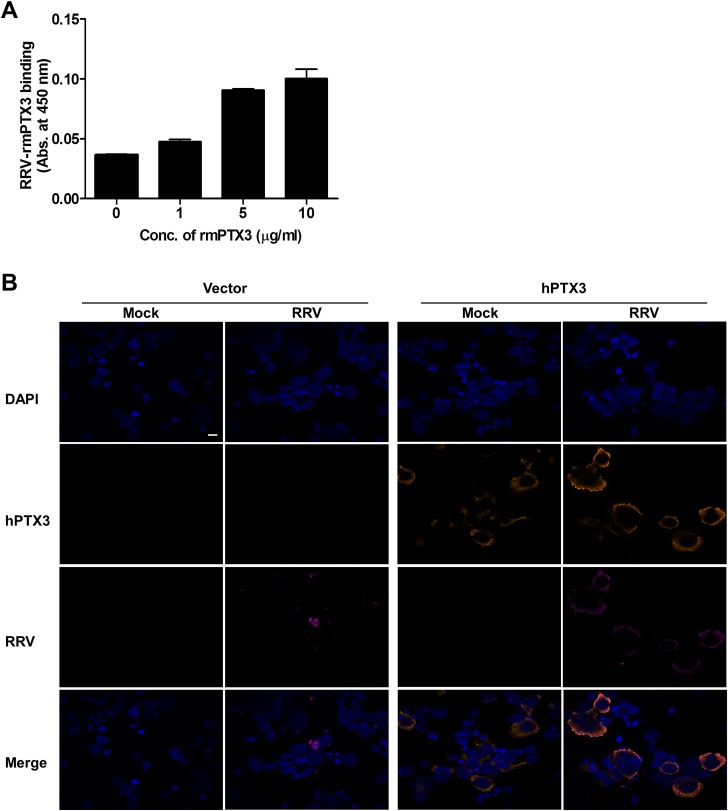
PTX3 binds to RRV and colocalizes in the cytoplasm during infection. (A) Different concentrations of mouse recombinant PTX3 were added to RRV-coated plates for 2 hours at 37°C, followed by binding to biotin-conjugated anti-PTX3 antibody for an additional 2 hours at 37°C. Optical density at 450 nm was read using Horseradish Peroxidase Substrate kit. (B) Vector- and hPTX3-transfected HEK293T cells were fixed at 6 hpi and stained for PTX3 (orange), RRV (magenta) and DAPI. Images are representative of 2 independent experiments. Magnification, ×60. Scale bar, 10 μm.

Previous studies have demonstrated that PTX3 binds to a range of microbes, including viruses. For cytomegalovirus [29] and influenza virus [30], recognition by PTX3 was shown to neutralize virus infectivity. To test whether PTX3 can bind to RRV, a microtitre plate-binding assay was performed. Microtitre wells coated with RRV were incubated with increasing concentrations of recombinant mouse PTX3 (rmPTX3) and RRV-PTX3 binding was determined. As seen in Fig. 9A, PTX3 bound to RRV in a dose-dependent manner. Similarly, a microtitre plate binding assay performed on CHIKV also demonstrated that PTX3 bound to CHIKV dose-dependently (S6D Fig.). Next, we examined whether PTX3 colocalizes with RRV during infection. During RRV infection of PTX3-overexpressing HEK 293T cells, RRV colocalized with PTX3 in the cytoplasm at 24 hpi (Fig. 9B). Similarly, RRV infection of HeLa cells, which are highly permissive to RRV infection and express endogenous PTX3 (S7A Fig.), demonstrated clear evidence of PTX3 colocalization with RRV in the cytoplasm during infection (S7B Fig.). These data show that during acute RRV infection, PTX3 forms a complex with RRV and colocalizes in the cytoplasm of the host cells, which may facilitate viral entry and replication processes.

### RRV infectivity is not affected in the presence of another acute phase protein—MBL

**Fig 10:**
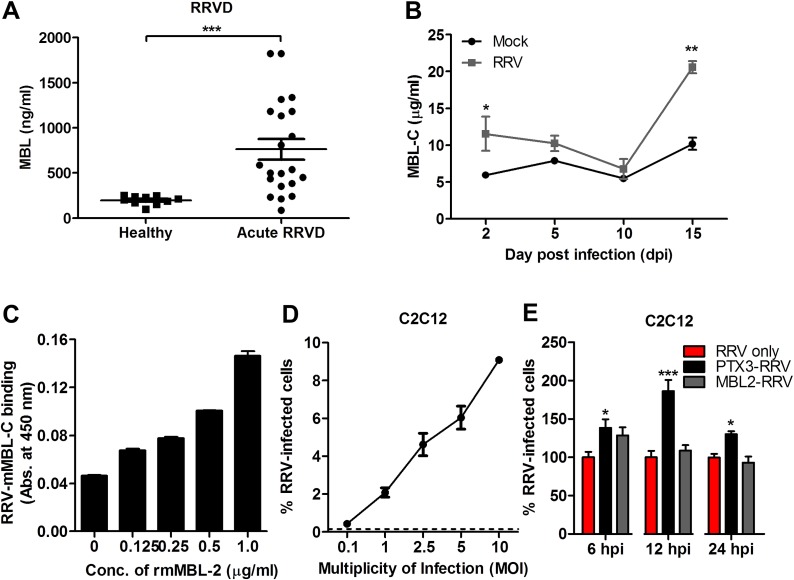
N-terminal of PTX3 is essential for binding to RRV and facilitates viral entry. (A) Schematic representation of structural features of human full-length (FL), N-terminal (N-term) and C-terminal (C-term) PTX3. (B) Different concentrations of human recombinant FL-PTX3, or (C) 5 μg/ml of human recombinant FL-, N-term- and C-term-PTX3, were added to RRV-coated plates for 2 hours at 37°C, followed by binding to biotin-conjugated anti-PTX3 antibody for additional 2 hours at 37°C. Optical density at 450 nm was read using Horseradish Peroxidase Substrate kit. Data are expressed as mean ± SEM of percent binding relative to FL-hPTX3 (*n* = 4). (D) PTX3-RRV complex-infected HEK293T cells were harvested at 0 and 6 hpi. Virus entry was quantified by flow cytometry using anti-alphavirus antibody. Data (*n* = 6) are presented as mean ± SEM and are representative of 2 independent experiments. **P < 0.01, ***P < 0.001, one-way ANOVA, Bonferroni’s post-test. (E) HEK293T cells were infected with RRV (10^4^ PFU RRV) and pre-bound PTX3-RRV complex (5 μg/ml of human recombinant FL-, N-term- or C-term-PTX3 + 10^4^ PFU RRV) for 24 hours. Supernatant was harvested and RRV titres was determined by plaque assay on Vero cells.

To confirm that the enhanced infectivity observed during acute RRV infection is specific to PTX3 and not to other acute phase immune proteins, a separate experiment was performed using another acute phase protein—MBL. As previously reported, serum MBL expression was significantly elevated in patients suffering from acute RRVD when compared to healthy controls (Fig. 10A) [19]. In the acute RRVD mouse model, elevated serum MBL-C was seen at both 2 and 15 dpi (Fig. 10B). Using a microtitre binding assay, a clear dose-dependent binding interaction between RRV and MBL-C was observed (Fig. 10C). Next, we infected C2C12 cells (Fig. 10D) with either complexed PTX3-RRV or MBL-RRV in order to identify the specificity of acute phase immune proteins in enhancing RRV infectivity. Enhanced infectivity was observed in cells infected with PTX3-RRV complex at 6, 12 and 24 hpi; however, no significant difference in infectivity was observed between RRV- or MBL-C-RRV complex-infected cells (Fig. 10E).

### N-terminal domain of PTX3 binds to RRV and is associated with enhanced viral entry and replication

**Fig 11:**
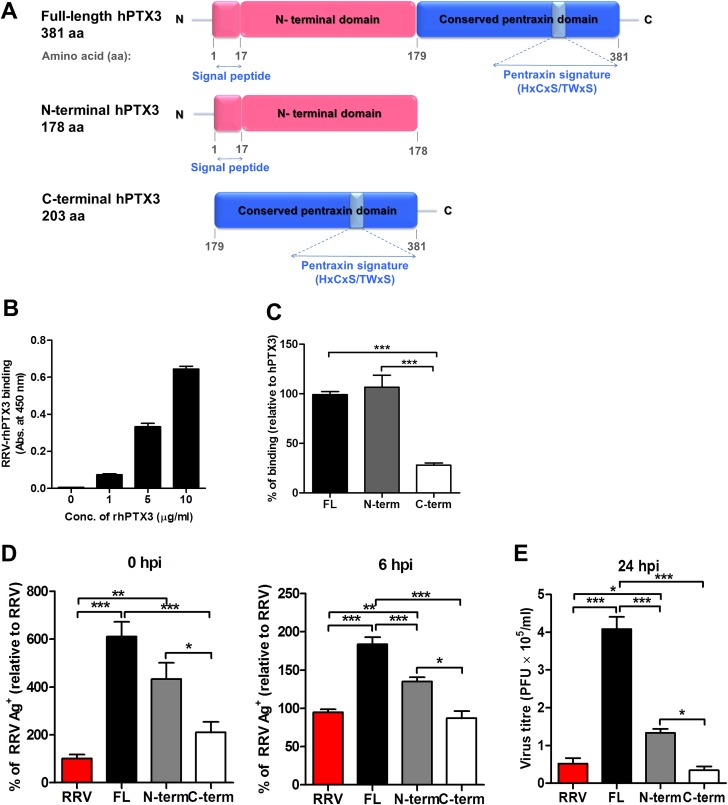
Acute phase protein MBL binds to RRV but does not affect viral infectivity. (A) Serum from RRVD patients (*n* = 21) or healthy controls (*n* = 10) were analyzed by ELISA for MBL levels. Data are presented as mean ± SEM. ***P < 0.001, Mann-Whitney *U* test. (B) 21-day-old C57BL/6 WT mice (*n* = 4–5 per group) were subcutaneously injected with 10^4^ PFU of RRV or PBS (mock). Mice were sacrificed at 2, 5, 10 and 15 dpi, and serum was collected for analysis of MBL-C expression by ELISA. Data are presented as mean ± SEM. *P < 0.05, **P < 0.01, two-way ANOVA, Bonferroni post-test. (C) Increasing concentrations of mouse recombinant MBL-C were added to RRV-coated plates for 2 hours at 37°C, followed by binding to biotin-conjugated anti-MBL-C antibody for additional 2 hours at 37°C. Optical density at 450 nm was read using Horseradish Peroxidase Substrate kit. (D) Dose-dependent infection of C2C12 cells was performed at MOI 0.1, 1, 2.5, 5 and 10 for 24 h, using EFGP-RRV. The percentage of infected cells (EGFP^+^) was assessed using flow cytometry analysis. (E) C2C12 cells were infected with EGFP-RRV (10^4^ PFU RRV) and pre-bound MBL-C-RRV or PTX3-RRV complex (1 μg/ml of mouse recombinant proteins + 10^4^ PFU RRV) for 6, 12 and 24 hours. The percentage of infected cells (EGFP^+^) was assessed using flow cytometry analysis. Horizontal dotted line represents the mean percentage of EGFP^+^ cells detected in mock control. *P < 0.05, ***P < 0.001, one-way ANOVA, Bonferroni’s post-test.

PTX3 consists of a conserved pentraxin C-terminal domain and a unique N-terminal domain. To determine the functional domain that is crucial for its functionality, we first examined the binding efficiency of recombinant human PTX3 (rhPTX3) N- and C-terminal fragments (Fig. 11A) to RRV. Full-length rhPTX3 bound to RRV in a dose-dependent manner (Fig. 11B) and the majority of binding activity could be mapped to the N-terminal domain. Removal of the N-terminal domain led to a significant reduction in RRV binding (Fig. 11C).

We next compared N- and C-terminal domains of rhPTX3 for their ability to facilitate RRV entry and replication. Briefly, RRV was pre-incubated with full-length rhPTX3, N-terminal-rhPTX3, or C-terminal-rhPTX3 and these mixtures were then added to HEK 293T cells. Examination of viral entry at 0 and 6 hpi revealed that N-terminal-rhPTX3 was approximately 30% less efficient in its ability to facilitate RRV entry, compared to full-length-rhPTX3. In contrast, removal of the N-terminal led to a complete ablation of PTX3-enhanced infection (Fig. 11D). Despite retaining approximately 70% of its ability to facilitate viral entry, infection of cells with RRV-N-terminal-rhPTX3 complex led to a reduced ability to enhance viral replication, compared to full-length rhPTX3. However, higher viral titre was still recovered from cells infected with RRV-N-terminal-rhPTX3 complex when compared to control infected with only RRV. No difference in viral titre was observed in cells infected with RRV-C-terminal-rhPTX3 complex (Fig. 11E).

Taken together, these data indicate that the N-terminal domain of PTX3 is responsible for the binding interaction with RRV and its functionality in facilitating viral entry.

## Discussion

Robust innate immune responses serve as the first line of host defense against alphavirus invasion. However, dysregulation of innate responses can also promote pathogenicity and disease. Consistent with this, we have previously identified overt expression of pro-inflammatory cytokines [37,38] and complement components [18] as pathogenic events in alphaviral diseases.

In the current study we sought to determine the role of PTX3, an acute phase protein associated with activation of the complement cascade [39], in the pathogenesis of alphaviral disease. During the acute phase of alphaviral infection, PTX3 was highly induced in serum and PBMCs of RRVD and CHIKF patients, respectively. Notably, the magnitude of PTX3 induction in CHIKF patients was dependent on viral load and disease severity. Similar observations have been reported for the short pentraxin C-reactive protein (CRP), which is a common laboratory marker for diagnosis of alphaviral infection [40,41]. Previously, Chow and colleagues reported that elevated expression of CRP was associated in CHIKF patients with high viral load and severe disease [15]. In addition to elevated PTX3 expression in alphavirus-infected patients, we also report abundant expression of PTX3 in serum and spleen of RRV-infected mice at the early stage of infection. During peak disease, PTX3 expression was also observed within the cellular infiltrates and further characterization identified inflammatory monocytes and neutrophils as the cellular sources of PTX3 during acute RRV infection. These findings indicate PTX3 is induced in response to alphaviral infections in humans and in mice. Elevated serum PTX3 expression has been observed in patients suffering from several arthritic conditions, including rheumatoid arthritis (2.08 ± 0.99 ng/ml), psoriatic arthritis (1.79 ± 0.80 ng/ml), polymyalgia rheumatic (2.08 ± 0.95 ng/ml), ankylosing spondylitis (2.48 ± 1.07 ng/ml) as well as other diseases such as giant cell arteritis (1.98 ± 1.05 ng/ml) and systemic lupus erythematosus (1.03 ± 0.84 ng/ml) [42]. Herein, the strong induction of PTX3 in RRVD (serum PTX3: 36.79 ± 8.443 ng/ml) and CHIKF patients suggests that PTX3 may also be included as a laboratory marker of acute alphaviral infection.

Dual roles of PTX3 have been reported in several pathogen-induced inflammatory diseases. Overexpression of PTX3 has protective effector function during bacterial infection with *Aspergillus fumigatus* [21,43], *Pseudomonas aeruginosa* [44] and uropathogenic *Escherichia coli* [45], as well as viral infections such as murine cytomegalovirus [29] and influenza virus [30]. Nevertheless, PTX3 expression has also been associated with exacerbated inflammatory responses and disease outcomes in intestinal ischemia-reperfusion injury [46] and pulmonary infection with *Klebsiella pneumonia* [47]. As PTX3 expression was associated with disease severity during acute alphaviral infections, we utilized an established RRVD mouse model [33] to examine the role of PTX3 during alphavirus infection. Deficiency of PTX3 was associated with delayed disease onset. While PTX3^-/-^ mice displayed similar clinical manifestations at peak of disease, these mice recovered more rapidly than WT animals. It has previously been reported that pro-inflammatory cytokines, including IFN-Ɣ, TNF-α and IL-6, and massive cellular infiltration contribute to inflammatory disease during alphaviral infections [37]. Indeed, delayed IFN-Ɣ, TNF-α and IL-6 responses were observed in quadricep muscles of PTX3^-/-^ mice during the peak of RRVD. In addition, PTX3^-/-^ mice showed diminished infiltration of inflammatory monocytes to the quadricep muscles during peak disease. Indeed, PTX3 has been shown to regulate leukocyte recruitment through interaction with P-selectin, leading to attenuation of cellular recruitment [32]. Using a peritoneal exudate model, we demonstrated increased recruitment of neutrophils and inflammatory monocytes in PTX3^-/-^ mice during early stages of infection. This observation may be associated with early upregulation of CCL2 and MIF, which are crucial for the recruitment of RRV-induced cellular infiltration [17,48] during early infection. PTX3 has been shown to bind apoptotic cells promoting deposition of complement components C3 and C1q [49]. Previously, it has been reported that C3 deposition during RRV infection contributes to the destruction of skeletal muscle tissues [18]. Hence, it is likely that the absence of PTX3 in our current study ameliorates complement-induced damage of muscle tissues in RRV-infected mice. Furthermore, we observed higher induction of iNOS in quadricep muscles of PTX3^-/-^ mice at peak RRVD. iNOS expression was recently shown to be pivotal in mediating skeletal muscle regeneration after acute damage [50]. These observations suggest PTX3 plays an immunomodulatory role during alphaviral infection. Moreover, the diminished infiltration of inflammatory monocytes and higher expression of iNOS during peak RRVD may contribute to rapid recovery from disease in the PTX3^-/-^ mice. Collectively, these data identify PTX3 as a pathogenic factor that shapes the progression of alphaviral disease through modulation of RRV-induced immune responses.

PTX3 is a pattern recognition molecule that interacts with viruses such as murine cytomegalovirus [29] and influenza virus [30], through which it can act to inhibit infection of target cells. In our study, *in vitro* and *in vivo* approaches were used to demonstrate that PTX3 promotes RRV infection and replication in host cells. Alphaviruses gain entry into host cells through receptor-mediated endocytosis, although the exact cell surface receptors involved remain poorly defined [51]. Herein, we demonstrate that both RRV and CHIKV can bind to PTX3. RRV and CHIKV infection of PTX3-expressing HEK 293T cells led to enhanced viral entry and replication. In addition, treatment of PTX3^-/-^ primary fibroblasts with rPTX3 also resulted in enhanced viral replication during early RRV infection, likely due to the formation of PTX3-RRV complex which enhances early viral entry events and replication. These data suggest that the extracellular interaction between PTX3 and RRV was involved in facilitating viral entry into host cells. The aggregates formed between RRV and PTX3 may promote more efficient multivalent binding to cell surface receptor/s for RRV, thereby promoting enhanced receptor-mediated endocytosis and viral entry. Alternatively, PTX3 may opsonize RRV and promote its uptake via putative (at this stage unknown) cell surface receptors for PTX3.

In addition to demonstrating the potential of PTX3 enhancing RRV entry into cells, we also report that the distribution of intracellular PTX3 was altered during RRV infection. Intracellular PTX3 migrates from perinuclear space to cytoplasm during infection and PTX3 co-localized with RRV in the cytoplasmic space suggests the possibility of intracellular associations between PTX3 and RRV. These interactions may further promote productive viral infection, perhaps by enhancing genomic replication. Indeed, we demonstrated that cells co-transfected with PTX3 and RRV, and harvested prior to the release of new virions had elevated levels of intracellular virus antigen. This result further supports the hypothesis that intracellular associations of PTX3 and RRV may promote viral replication processes. Moreover, the presence of PTX3 was crucial for enhanced viral replication during RRV infection of WT mice and PTX3-overexpressing HEK 293T cells. Together, this study shows that PTX3-RRV interaction gives rise to pathogenic effect, enhancing viral entry and replication, in contrast to previous studies using other viruses such as murine cytomegalovirus [29] and influenza virus [30], where PTX3 binding was associated with virus neutralization, thereby contributing to a protective host response.

PTX3 is a structurally complex multimeric protein, comprising a highly conserved C-terminal domain shared across all members of the pentraxin family and a unique N-terminal domain whose structure is poorly characterized. We showed that the N-terminal domain is crucial for PTX3 binding to RRV and PTX3-mediated enhancement of RRV infection. However, removal of the C-terminal domain did affect the ability of the N-terminal domain of PTX3 to modulate viral replication, resulting in only partial enhancement of viral replication compared to full-length PTX3. Previous studies have reported the importance of an intact quaternary structure in order for PTX3 to retain its binding and biological efficacies [52]. Therefore, full-length PTX3 with intact quaternary structure would be necessary to retain its biological role of enhancing RRV replication.

Taken together, the data presented in this study provides the first evidence of a role for PTX3 in enhancing RRV uptake and replication during early alphaviral infection. PTX3 has previously been associated with protective functions against a number of viruses, including influenza virus [30], human/murine cytomegalovirus [7] and coronavirus murine hepatitis virus [53], in contrast to the pathogenic role identified in the current study. Our findings demonstrate a previously undescribed pivotal role of PTX3 in shaping alphaviral disease progression through immunomodulation and facilitating viral infection and replication processes during the acute phase of infection. In conclusion, our findings provide new insight into the role of PTX3 in acute alphaviral infection. The newly identified role of PTX3 in enhancing RRV infection and replication also sheds light on the poorly defined route of alphavirus entry into host cells. Given the diverse functional roles of PTX3 as well as its ability to bind to a variety of immune factors, further study is required to define the exact PTX3-triggered immune pathways induced in alphaviral-induced arthritic diseases. Identification of such pathways will be an important step towards the future development of therapeutic interventions.

## Materials and Methods

### Ethics statement

Animal experiments were approved by the Animal Ethics Committee of Griffith University (BDD/01/11/AEC). All procedures involving animals conformed to the National Health and Medical Research Council Australian code of practice for the care and use of animals for scientific purposes 8th edition 2013. CHIKV human PBMC samples were collected from 20 patients that were admitted to the Communicable Disease Centre at Tan Tock Seng Hospital during the 2008 Singapore CHIKF outbreak. All patients were diagnosed with CHIKF and blood were collected at the acute phase (median of 4 days after illness onset) of infection [54], with written informed consent obtained from all participants. The study was approved by the National Healthcare Group’s domain-specific ethics review board (DSRB Reference No. B/08/026). All RRV human serum samples had been submitted to the Centre for Infectious Diseases and Microbiology Laboratory Services (CIDMLS), Westmead Hospital for diagnostic testing and laboratory investigation of RRV with written and oral informed patient consent. Serum from healthy individuals was provided by Australian Red Cross with written and oral informed consent, approved by Griffith University Human Research Ethics Committee (BDD/01/12/HREC). No new human samples were collected as part of this study. Serum samples were de-identified before being used in the research project.

### Patients

PBMC specimens of 20 patients were classified into viral load (high viral load, HVL; *n* = 10 and low viral load, LVL; *n* = 10) and disease severity (severe illness; *n* = 10 and mild illness; *n* = 10) groups, as described previously [15]. Briefly, the HVL and LVL groups had mean viral loads of 1.31 × 10^8^ PFU/ml and 1.95 × 10^4^ PFU/ml respectively, while severe illness were defined as having a temperature of higher than 38.5°C, pulse rate more than 100 beats/min or platelet count less than 100 × 10^9^ cells/L. Serum specimens were collected from 21 acute cases of RRV-induced polyarthritis patients in Australia. PBMCs and serum specimens isolated from 10 healthy volunteers were used as controls. All specimens were stored at -80°C until use.

### Virus

Stocks of the WT T48 strain of RRV were generated from the full-length T48 cDNA clone, kindly provided by Richard Kuhn, Purdue University, West Lafayette, IN. The CHIKV variant expressing mCherry (CHIKV-mCherry) was constructed using a full-length infectious cDNA clone of the La Reunion CHIKV isolate LR2006-OPY1 as described previously [55].

### Cell culture, proteins and transfection

HEK 293T, HeLa and C2C12 cells were cultured in DMEM supplemented with 10% FBS. Primary fibroblasts were isolated from tails of WT and PTX3^-/-^ mice using a previously described protocol [56] and cultured in DMEM supplemented with 20% FCS. Transient transfection of PTX3 plasmids [57] was performed using Lipofectamine 2000 (Invitrogen) following manufacturer’s instructions. Electroporation of RRV T48 infectious plasmid clone [33] was performed using Eppendorf Eporator. Recombinant N-terminal and C-terminal PTX3 proteins were purified as described in [58]. Recombinant mouse and human PTX3, and mouse MBL-C were purchased from R&D.

### *In vitro* RRV or CHIKV infection

HEK 293T cells and primary tail fibroblasts were plated at a density of 1.0 × 10^5^ per well on 24-well plates overnight, prior to infection with RRV or CHIKV (MOI 1) for 1 h at 37°C in humidified CO_2_ incubator. Virus overlay was removed and 1 ml of pre-warmed growth medium was added to the monolayer of cells, marking the 0 hour post infection (hpi). Cells were incubated at 37°C in humidified CO_2_ incubator and were harvested accordingly.

### Virus plaque assay

All titrations were performed by plaque assay on Vero cells as described previously [59].

### Microtitre plate binding assay using immobilized viruses

Microtiter plates (Sarstedt) were coated overnight at 4°C with 0.1M carbonate-bicarbonate coating buffer alone or containing either 10^4^ PFU RRV or CHIKV (UV-inactivated for 30 min). Non-specific binding sites were blocked by 5% BSA in PBS for 1 h at room temperature. Recombinant PTX3 or MBL-C binding to virus was performed by incubating recombinant proteins on virus-coated microtitre plate for 2 h at 37°C. Biotin-conjugated anti-PTX3 or anti-MBL-C detection antibody (R&D) was added and incubated at room temperature for 2 h. The optical density at 450 nm was read using the streptavidin conjugated to horseradish-peroxidase (HRP) substrate (R&D).

### Total RNA extraction and cDNA synthesis

Total RNA extraction was performed using TRIzol reagent (Life Technologies) following manufacturer’s instructions. Quantification of total RNA was measured by NanoDrop 1000 spectrophotometer (Thermo Scientific). Extracted total RNA (10 ng/μL) was reverse-transcribed using an oligo (dT) primer and M-MLV reverse transcriptase (Sigma Aldrich) according to the manufacturer’s instructions.

### Gene expression qRT-PCR

qRT-PCR was performed using SsoAdvanced Universal SYBR Green Supermix (BIO-RAD) in 12.5 μl of reaction volume. Reactions were performed using QuantiTect Primer Assay kits (Qiagen) and BIO-RAD CFX96 Touch Real-Time PCR Detection System on 96-well plates. Cycler conditions were as follows: (i) PCR initial activation step: 95°C for 15 min, 1 cycle and (ii) 3-step cycling: 94°C for 15 sec, follow by 55°C for 30 sec and 72°C for 30 sec, 40 cycles. Dissociation curves for each gene were acquired using CFX Manager software to determine specificity of amplified products. The fold change relative to healthy donors/mock samples for each gene was calculated with the ΔΔ*Ct* method using Microsoft Excel 2010. Briefly, ΔΔ*Ct* = Δ*Ct*(patient/infected)–Δ*Ct*(healthy donor/mock) with Δ*Ct* = *Ct*(gene-of-interest)—*Ct*(housekeeping gene-GAPDH/HPRT). The fold change for each gene is calculated as 2^-ΔΔ*Ct*^.

### Viral load qRT-PCR

Standard curve was generated using serial dilutions of RRV T48 infectious plasmid DNA as described previously [33]. Quantification of viral load was performed using SsoAdvanced Universal Probes Supermix (BIO-RAD) in 12.5 μl reaction volume to detect nsP3 region RNA, using specific probe (5-ATTAAGAGTGTAGCCATCC-3’) and primers (forward: 5’-CCGTGGCGGGTATTATCAAT-3’; reverse: 5’-AACACTCCCGTCGACAACAGA-3’)[60]. Reactions were performed using BIO-RAD CFX96 Touch Real-Time PCR Detection System on 96-well plates. Cycler conditions were as follows: (i) PCR initial activation step: 95°C for 3 min, 1 cycle and (ii) 2-step cycling: 95°C for 15 sec, followed by 60°C for 45 sec, 45 cycles. Standard curve was plotted and copy numbers of amplified products were interpolated from standard curve using Prism Graphpad software to determine viral load.

### Immunofluorescence staining

Transfected HEK 293T cells were seeded on poly-L-lysine-coated coverslips for staining. Cells were fixed with 2% paraformaldehyde (PFA), permeabilized in PBS containing 0.1% Triton X-100, and blocked with 20% goat serum in PBS. Cells were incubated with rat monoclonal anti-PTX3 (MNB4, Abcam) or mouse monoclonal anti-alphavirus (3581, Santa Cruz) primary antibody in PBS, followed by goat anti-rat AF488 (Invitrogen) or goat anti-mouse AF647 (Invitrogen) secondary antibody. Cells were washed, mounted, and examined with a confocal laser-scanning microscope (Fluoview FV 1000, Olympus) at 60x magnification. Images were collected and processed using FV1000-ASW software.

### ELISA

ELISAs were performed using DuoSet ELISA Development kit (R&D systems) following manufacturer’s instructions.

### Flow cytometry

To analyze PTX3 intracellular expression, transfected HEK 293T cells were fixed with 2% PFA and permeabilized with 0.1% Saponin (Sigma Aldrich) in PBS. Indirect intracellular staining was performed with rat anti-PTX3 (MNB4, Abcam) primary antibody, followed by AF488-conjugated anti-rat (Life Technologies) secondary antibody. To identify the various cell populations present in splenocytes, peritoneal lavage and quadriceps harvested from mice, cells were first incubated with anti-mouse CD16 / CD32 (FC block, BD Pharmingen) and stained with the following antibodies: APC-conjugated anti-mouse GR1, PE-conjugated anti-mouse F4/80, FITC-conjugated anti-mouse CD11b, APC-conjugated anti-mouse Ly6c, APC-conjugated anti-mouse CD3, FITC-conjugated anti-mouse CD19, PE-conjugated anti-mouse CD45, or PE-Cy7-conjugated anti-mouse NK1.1 (BD Pharmingen). For detection of alphavirus antigens, indirect intracellular staining was performed using mouse monoclonal anti-alphavirus (3581, Santa Cruz) primary antibody, followed by AF488-conjugated anti-mouse (Life Technologies) secondary antibody. Data acquisition was performed using CyanADP (Beckman Coulter), and analysis was done by Kaluza Flow Analysis Software (Beckman Coulter).

### Animal studies

6–8 week-old C57BL/6 male and female mice, of equal distribution, were inoculated intraperitoneally with 10^5^ PFU RRV in 500 μl of PBS, to study the early effect of PTX3 deficiency on recruitment of neutrophils and inflammatory monocytes. Peritoneal lavage was harvested at 6 hpi with 5 ml of ice-cold PBS.

For the acute RRV mouse model, 21-day-old C57BL/6 male and female mice, of equal distribution, were inoculated subcutaneously in the thorax below the right forelimb with 10^4^ PFU RRV in 50 μl. Mock-infected mice were inoculated with PBS diluent alone. Mice were weighed and scored for disease signs every 24 h. Mice were assessed based on animal strength and hind-leg paralysis, as outlined previously [33], using the following scale: 0, no disease signs; 1, ruffled fur; 2, very mild hindlimb weakness; 3, mild hindlimb weakness; 4, moderate hindlimb weakness; 5, severe hindlimb weakness, 6, complete loss of hindlimb function; and 7, moribund. Mice were euthanized, quadriceps and ankle joints were removed and homogenized using QIAGEN Tissuelyser II then centrifuged at 12, 000 × g, 5 min, 4°C. Blood was collected via cardiac puncture. Serum was isolated by centrifugation at 12, 000 × g, 5 min, 4°C. For analysis of infiltrating inflammatory cells by flow cytometry, mice were sacrificed and perfused with PBS at 7 dpi. Quadricep muscles were harvested, weighed, minced, and digested with DMEM containing 20% FBS, 1 mg/ml of collagenase IV (Roche) and 1 mg/ml of DNase I (Roche), for 1 h at 37°C. Cells were strained through a 40 μm strainer (BD Biosciences) and washed with DMEM containing 20% FBS and viable cells were counted by trypan blue exclusion.

For histology, quadricep muscles harvested were fixed in 4% PFA, followed by paraffin-embedding. Five-micrometer sections were prepared. IHC was performed on dewaxed, rehydrated, 5 μm paraffin-embedded tissue sections. Sections were incubated with 20% goat serum (Gibco) in 5% BSA/PBS for 20 min. Primary antibody staining was performed using rat anti-mouse PTX3 (MNB1, Abcam) in PBS, incubated overnight, at 4°C, in humidified chamber. Tissue sections were washed in PBS for three times at 5 min intervals. Secondary antibody staining was performed using HRP-conjugated anti-rat IgG2b (Serotec) incubated for 30 min, room temperature, in a humidified chamber. Colour was developed with 3,3’-diaminobenzidine (DAB) Peroxidase Substrate Kit (Vector Laboratories), according to manufacturer’s instructions and counter-stained with hematoxylin (Vector Laboratories).

### Statistics

All statistical analyses were performed using Prism 5.01 (Graph-Pad Software). Analysis of PTX3 expression profiles in comparison between healthy and RRVD or CHIKF patients, HVL and LVL CHIKF patients’ groups, and severe and mild illness CHIKF patients’ groups was done using Mann-Whitney *U* test. Comparisons of PTX3 expression among different time points post infection in WT mice, PTX3 expression in mock- and RRV-infected mouse splenocytes, clinical scoring of between PTX3^-/-^ and WT mice, viral replication and viral entry among RRV-infected HEK 293T cells and fibroblasts, were performed using two-way ANOVA with Bonferroni post-test. Comparisons of viral replication and viral entry among RRV-, FL-PTX3, N-term-PTX3 and C-term-PTX3-RRV infected groups were analyzed using one-way ANOVA with Bonferroni post-test. Analyses of all other experimental groups were performed using student unpaired *t*-test. *P* values less than 0.05 were considered statistically significant.

## Supporting Information

S1 Fig PTX3 deficiency leads to reduced viral load in ankle joints. 21-day-old C57BL/6 WT and PTX3^-/-^ mice were infected subcutaneously with 10^4^ PFU RRV at the thorax region. Viral load in ankle joint and quadriceps of RRV-infected WT and PTX3^-/-^ mice (*n* = 3–7 per group) at (A) 2 and (B) 10 dpi were determined using TaqMan qRT-PCR with specific probe and primers against RRV nsP3 RNA. Data are presented as mean ± SEM. *P < 0.05, Student unpaired *t*-test.
(TIF)

S2 Fig CCL2 and MIF are up-regulated during early RRV infection in mice. 21-day-old C57BL/6 WT and PTX3^-/-^ (*n* = 4–7 per group) mice were infected subcutaneously with 10^4^ PFU RRV. Transcriptional profiles of immune mediators, (A) CCL2, (B) MIF, (C) CCL3, (D) CXCL1 and (E) CXCL2 were determined by qRT-PCR, from the quadriceps at early RRV disease (2 dpi) and peak RRV disease (10 dpi). Data were normalized to *HPRT* and are shown as fold expression relative to WT. Data are presented as mean ± SEM. *P < 0.05, Student unpaired *t*-test.
(TIF)

S3 Fig PTX3 expression in HEK 293T cells. HEK293T cells were transfected with vector plasmid for 20 h before RRV infection at MOI 1 for 24 h. Cells were fixed at 6 hpi and stained for PTX3 (green) and DAPI (blue). Images are representative of 2 independent experiments. Magnification, ×60. Scale bar, 10 μm.
(TIF)

S4 Fig PTX3-expressing HEK293T cells exhibit higher viral load during early hours of RRV infection. HEK293T cells were transfected with human PTX3 or vector plasmid for 20 h before RRV infection. (A) Dose-dependent infection of transfected HEK 293T cells was performed at MOI 0.1, 0.5, 1, 2.5 and 5 for 24 h. Supernatants were harvested and RRV titres determined by plaque assay. (B) Transfected HEK293T cells were infected at MOI 1. Cells were harvested at 0, 1, 2, 3, 4, 5 and 6 hpi for viral load analysis, determined using TaqMan qRT-PCR with specific probe and primers against RRV nsP3 RNA. Data are presented as mean ± SEM. *P < 0.05, Student unpaired *t*-test.
(TIF)

S5 Fig Flow cytometry analysis of RRV-positive HEK 293T cells co-expressing PTX3. HEK293T cells were transfected with human PTX3 or vector plasmid for 20 h before RRV infection. Transfected HEK293T cells were harvested at 0 and 6 hpi to assess for intracellular RRV and PTX3 expression using flow cytometry analysis.
(TIF)

S6 Fig PTX3 binds to CHIKV and enhances viral entry and replication. (A) HEK293T cells were transfected with human PTX3 or vector plasmid for 20 h before CHIKV infection at MOI 1 for 24 h. Supernatants were harvested and CHIKV titres were determined by plaque assay. Data are presented as mean ± SEM. **P < 0.005, Student unpaired *t*-test. Transfected HEK293T cells were harvested at 0 and 6 hpi, (B) to assess intracellular CHIKV expression by flow cytometry using anti-alphavirus antibody for detection of viral entry, and (C) to assess intracellular PTX3 expression using flow cytometry analysis. Data (*n* = 3) are presented as mean ± SEM and are representative of 2 independent experiments. *P < 0.05 **P < 0.005, ***P < 0.001, two-way ANOVA, Bonferroni post-test. (D) Different concentrations of mouse recombinant PTX3 were added to CHIKV-coated plate for 2 hours at 37°C, followed by binding to biotin-conjugated anti-PTX3 antibody for an additional 2 hours at 37°C. Optical density at 450 nm was read using Horseradish Peroxidase Substrate kit.
(TIF)

S7 Fig HeLa cells express PTX3, which colocalizes with RRV in the cytoplasm during infection. (A) Dose-dependent infection of HeLa cells with RRV were performed at MOI 0.1, 0.5 and 1 for 24 h. Supernatants were harvested and RRV titres determined by plaque assay on Vero cells. Data are presented as mean ± SEM. (B) HeLa cells were infected with RRV (MOI 1) and cells were harvested at 24 hpi, fixed and stained for PTX3 (green), RRV (red) and DAPI (blue). Images are representative of 2 independent experiments. Magnification, ×60. Scale bar, 10 μm.
(TIF)
