# Correction: Role of Pentraxin 3 in Shaping Arthritogenic Alphaviral Disease: From Enhanced Viral Replication to Immunomodulation

**DOI:** 10.1371/journal.ppat.1004797

**Published:** 2015-04-13

**Authors:** The PLOS Pathogens Staff

The figure legends for Figs. 10 and 11 are swapped. The legends for Fig. 9 and S3 Fig. incorrectly state that cells were fixed at 6 hpi. The correct figure legends should state they were fixed at 24 hpi. The correct legends for all four figures are provided below.

**Fig 9. PTX3 binds to RRV and colocalizes in the cytoplasm during infection.** (A) Different concentrations of mouse recombinant PTX3 were added to RRV-coated plates for 2 hours at 37°C, followed by binding to biotin-conjugated anti-PTX3 antibody for an additional 2 hours at 37°C. Optical density at 450 nm was read using Horseradish Peroxidase Substrate kit. (B) Vector- and hPTX3-transfected HEK293T cells were fixed at 24 hpi and stained for PTX3 (orange), RRV (magenta) and DAPI. Images are representative of 2 independent experiments. Magnification, ×60. Scale bar, 10 μm.

**Fig 10. Acute phase protein MBL binds to RRV but does not affect viral infectivity.** (A) Serum from RRVD patients (n = 21) or healthy controls (n = 10) were analyzed by ELISA for MBL levels. Data are presented as mean ± SEM. ***P < 0.001, Mann-Whitney U test. (B) 21-day-old C57BL/6 WT mice (n = 4–5 per group) were subcutaneously injected with 104 PFU of RRV or PBS (mock). Mice were sacrificed at 2, 5, 10 and 15 dpi, and serum was collected for analysis of MBL-C expression by ELISA. Data are presented as mean ± SEM. *P < 0.05, **P < 0.01, two-way ANOVA, Bonferroni post-test. (C) Increasing concentrations of mouse recombinant MBL-C were added to RRV-coated plates for 2 hours at 37°C, followed by binding to biotin-conjugated anti-MBL-C antibody for additional 2 hours at 37°C. Optical density at 450 nm was read using Horseradish Peroxidase Substrate kit. (D) Dose-dependent infection of C2C12 cells was performed at MOI 0.1, 1, 2.5, 5 and 10 for 24 h, using EFGP-RRV. The percentage of infected cells (EGFP+) was assessed using flow cytometry analysis. (E) C2C12 cells were infected with EGFP-RRV (104 PFU RRV) and pre-bound MBL-C-RRV or PTX3-RRV complex (1 μg/ml of mouse recombinant proteins + 104 PFU RRV) for 6, 12 and 24 hours. The percentage of infected cells (EGFP+) was assessed using flow cytometry analysis. Horizontal dotted line represents the mean percentage of EGFP+ cells detected in mock control. *P < 0.05, ***P < 0.001, one-way ANOVA, Bonferroni’s post-test.

**Fig 11. N-terminal of PTX3 is essential for binding to RRV and facilitates viral entry.** (A) Schematic representation of structural features of human full-length (FL), N-terminal (N-term) and C-terminal (C-term) PTX3. (B) Different concentrations of human recombinant FL-PTX3, or (C) 5 μg/ml of human recombinant FL-, N-term- and C-term-PTX3, were added to RRV-coated plates for 2 hours at 37°C, followed by binding to biotin-conjugated anti-PTX3 antibody for additional 2 hours at 37°C. Optical density at 450 nm was read using Horseradish Peroxidase Substrate kit. Data are expressed as mean ± SEM of percent binding relative to FL-hPTX3 (n = 4). (D) PTX3-RRV complex-infected HEK293T cells were harvested at 0 and 6 hpi. Virus entry was quantified by flow cytometry using anti-alphavirus antibody. Data (n = 6) are presented as mean ± SEM and are representative of 2 independent experiments. **P < 0.01, ***P < 0.001, one-way ANOVA, Bonferroni’s post-test. (E) HEK293T cells were infected with RRV (104 PFU RRV) and pre-bound PTX3-RRV complex (5 μg/ml of human recombinant FL-, N-term- or C-term-PTX3 + 104 PFU RRV) for 24 hours. Supernatant was harvested and RRV titre was determined by plaque assay on Vero cells.

**S3 Fig. PTX3 expression in HEK 293T cells.** HEK293T cells were transfected with vector plasmid for 20 h before RRV infection at MOI 1 for 24 h. Cells were fixed at 24 hpi and stained for PTX3 (green) and DAPI (blue). Images are representative of 2 independent experiments. Magnification, ×60. Scale bar, 10 μm.
