# Supplementary figures and images for: Role of Pentraxin 3 in Shaping Arthritogenic Alphaviral Disease: From Enhanced Viral Replication to Immunomodulation
Source: PLoS Pathog. 2015 Feb 19;11(2):e1004649. doi: 10.1371/journal.ppat.1004649 (PMC4335073; doi:10.1371/journal.ppat.1004649)

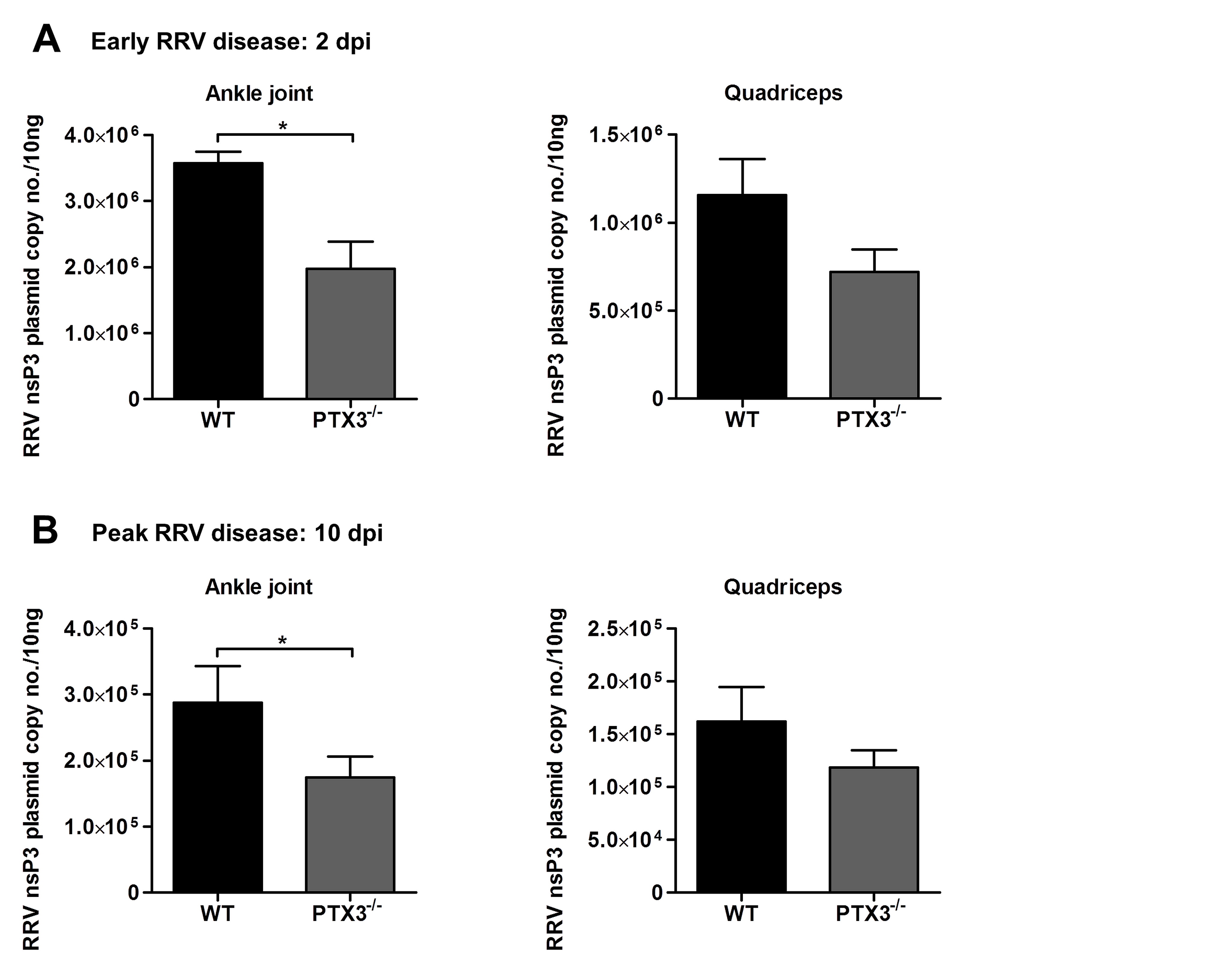

Supplement: S1 Fig — 21-day-old C57BL/6 WT and PTX3-/- mice were infected subcutaneously with 104 PFU RRV at the thorax region. Viral load in ankle joint and quadriceps of RRV-infected WT and PTX3-/- mice (n = 3–7 per group) at (A) 2 and (B) 10 dpi were determined using TaqMan qRT-PCR with specific probe and primers against RRV nsP3 RNA. Data are presented as mean ± SEM. *P < 0.05, Student unpaired t-test. (TIF) [file ppat.1004649.s001.tif]

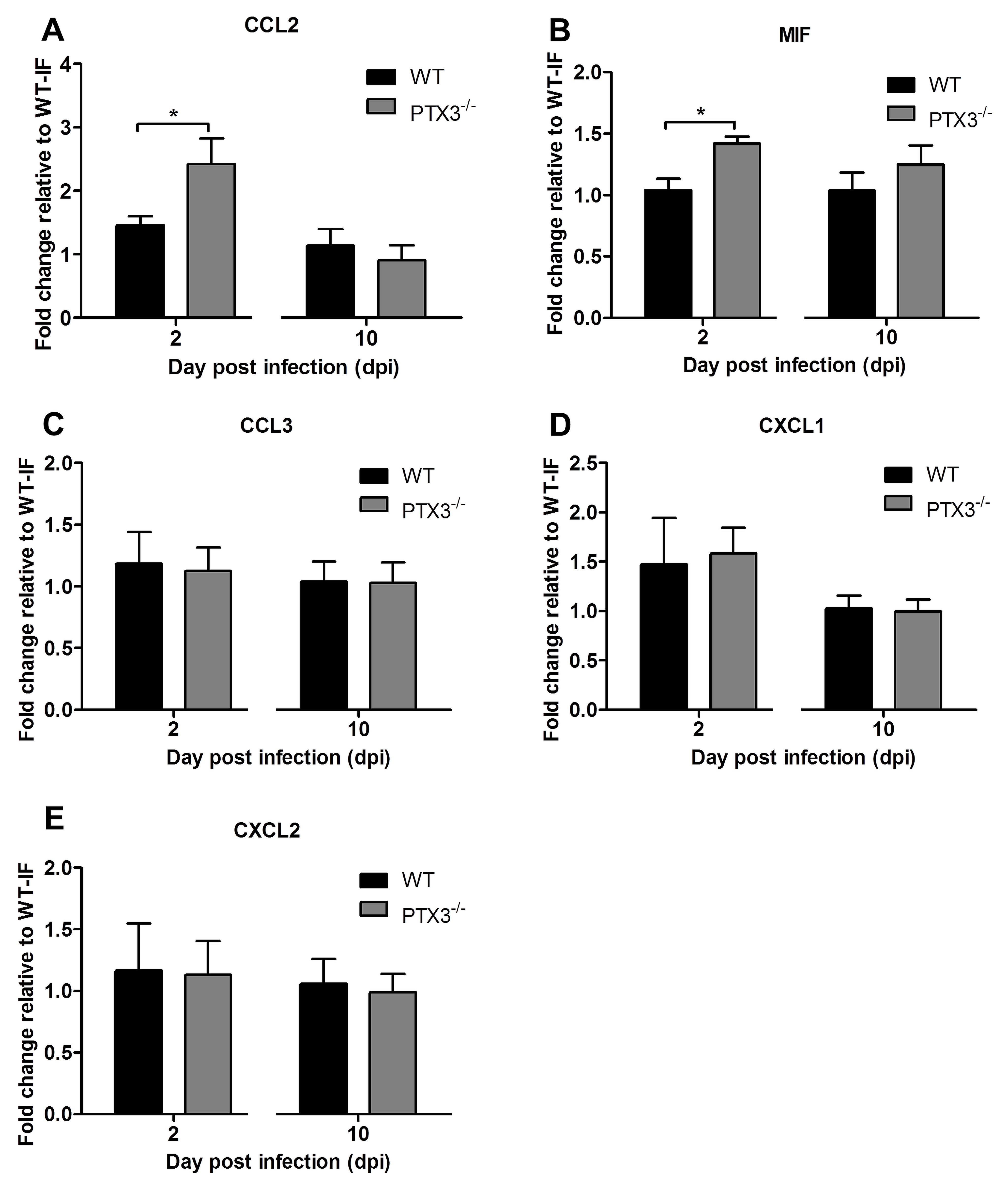

Supplement: S2 Fig — 21-day-old C57BL/6 WT and PTX3-/- (n = 4–7 per group) mice were infected subcutaneously with 104 PFU RRV. Transcriptional profiles of immune mediators, (A) CCL2, (B) MIF, (C) CCL3, (D) CXCL1 and (E) CXCL2 were determined by qRT-PCR, from the quadriceps at early RRV disease (2 dpi) and peak RRV disease (10 dpi). Data were normalized to HPRT and are shown as fold expression relative to WT. Data are presented as mean ± SEM. *P < 0.05, Student unpaired t-test. (TIF) [file ppat.1004649.s002.tif]

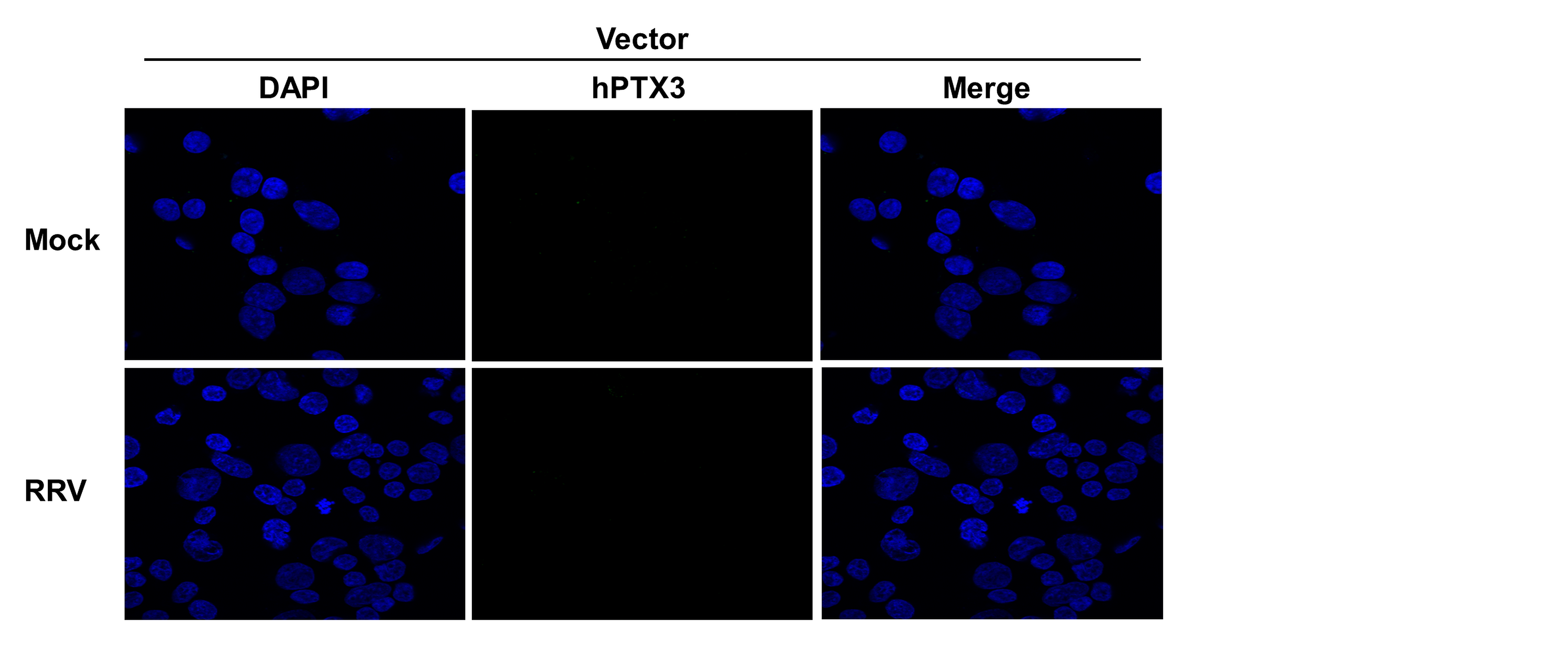

Supplement: S3 Fig — HEK293T cells were transfected with vector plasmid for 20 h before RRV infection at MOI 1 for 24 h. Cells were fixed at 6 hpi and stained for PTX3 (green) and DAPI (blue). Images are representative of 2 independent experiments. Magnification, ×60. Scale bar, 10 μm. (TIF) [file ppat.1004649.s003.tif]

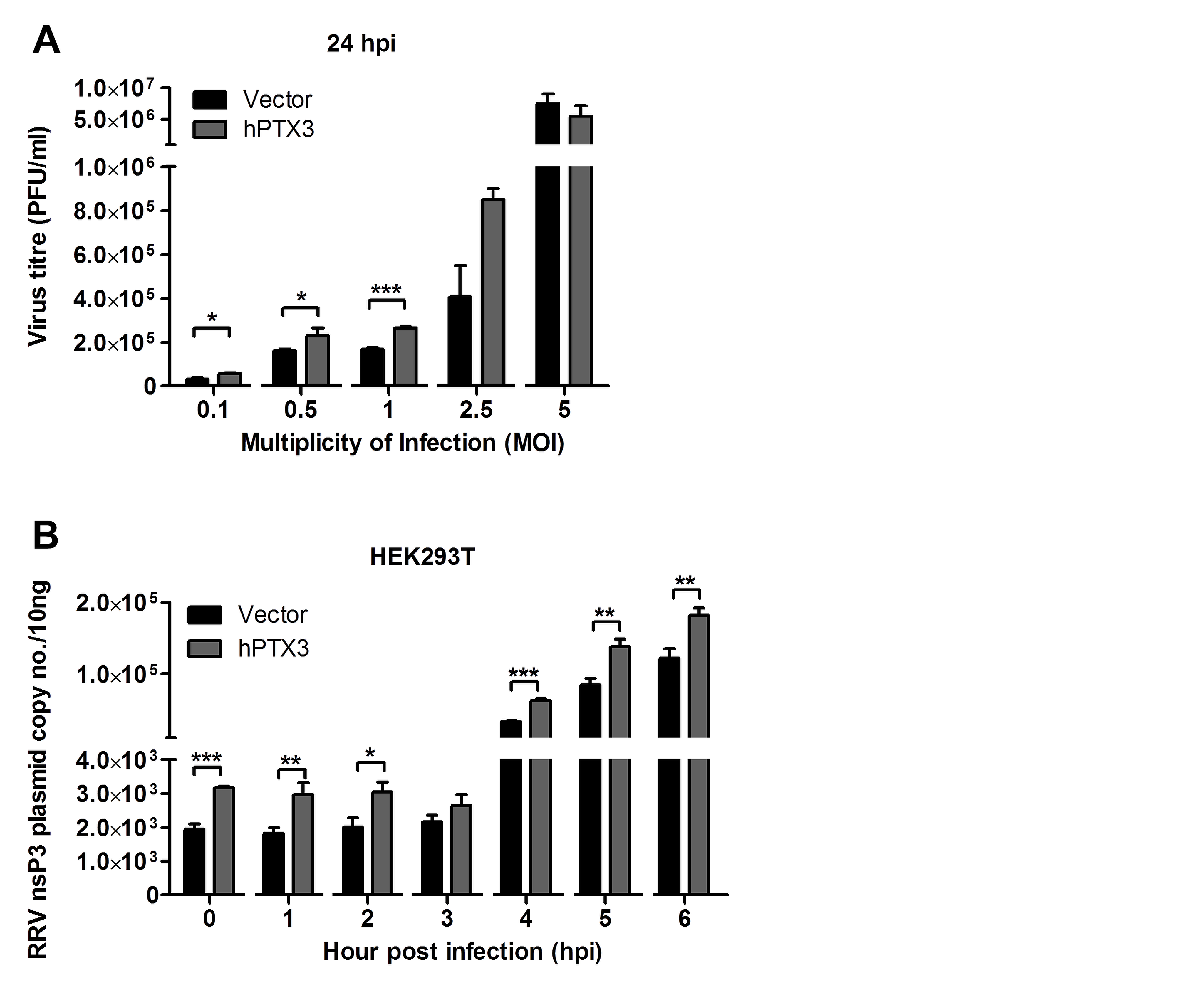

Supplement: S4 Fig — HEK293T cells were transfected with human PTX3 or vector plasmid for 20 h before RRV infection. (A) Dose-dependent infection of transfected HEK 293T cells was performed at MOI 0.1, 0.5, 1, 2.5 and 5 for 24 h. Supernatants were harvested and RRV titres determined by plaque assay. (B) Transfected HEK293T cells were infected at MOI 1. Cells were harvested at 0, 1, 2, 3, 4, 5 and 6 hpi for viral load analysis, determined using TaqMan qRT-PCR with specific probe and primers against RRV nsP3 RNA. Data are presented as mean ± SEM. *P < 0.05, Student unpaired t-test. (TIF) [file ppat.1004649.s004.tif]

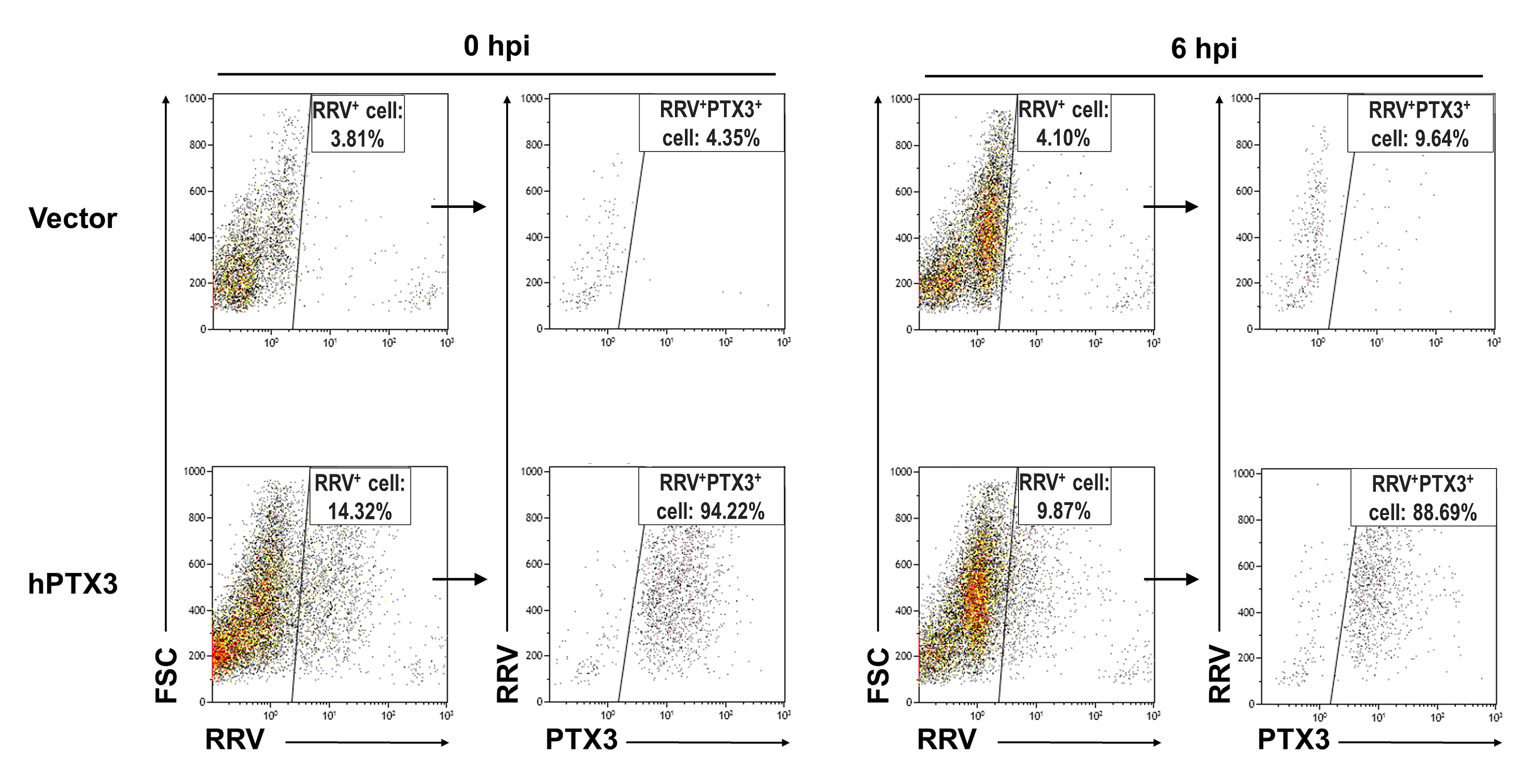

Supplement: S5 Fig — HEK293T cells were transfected with human PTX3 or vector plasmid for 20 h before RRV infection. Transfected HEK293T cells were harvested at 0 and 6 hpi to assess for intracellular RRV and PTX3 expression using flow cytometry analysis. (TIF) [file ppat.1004649.s005.tif]

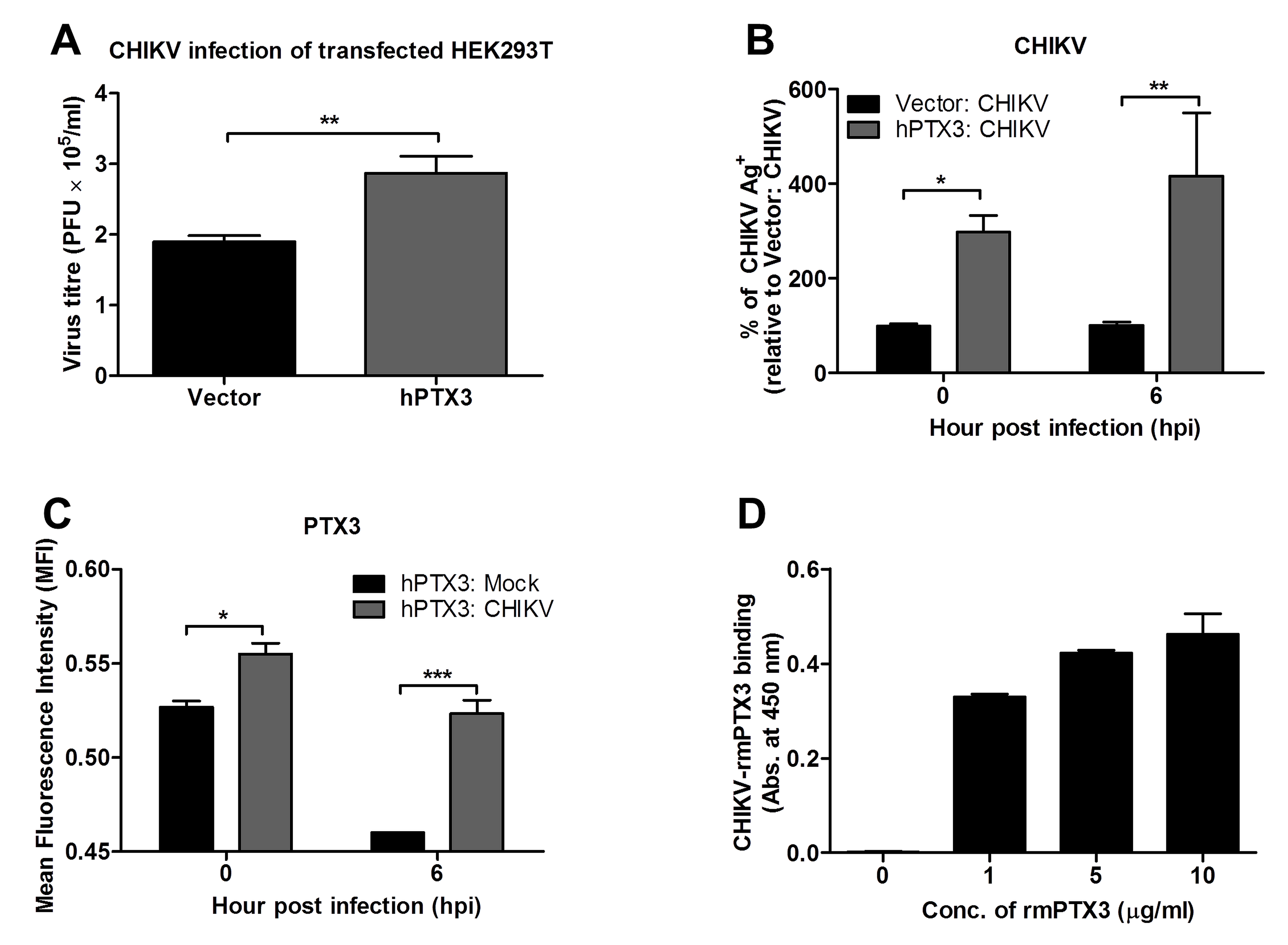

Supplement: S6 Fig — (A) HEK293T cells were transfected with human PTX3 or vector plasmid for 20 h before CHIKV infection at MOI 1 for 24 h. Supernatants were harvested and CHIKV titres were determined by plaque assay. Data are presented as mean ± SEM. **P < 0.005, Student unpaired t-test. Transfected HEK293T cells were harvested at 0 and 6 hpi, (B) to assess intracellular CHIKV expression by flow cytometry using anti-alphavirus antibody for detection of viral entry, and (C) to assess intracellular PTX3 expression using flow cytometry analysis. Data (n = 3) are presented as mean ± SEM and are representative of 2 independent experiments. *P < 0.05 **P < 0.005, ***P < 0.001, two-way ANOVA, Bonferroni post-test. (D) Different concentrations of mouse recombinant PTX3 were added to CHIKV-coated plate for 2 hours at 37°C, followed by binding to biotin-conjugated anti-PTX3 antibody for an additional 2 hours at 37°C. Optical density at 450 nm was read using Horseradish Peroxidase Substrate kit. (TIF) [file ppat.1004649.s006.tif]

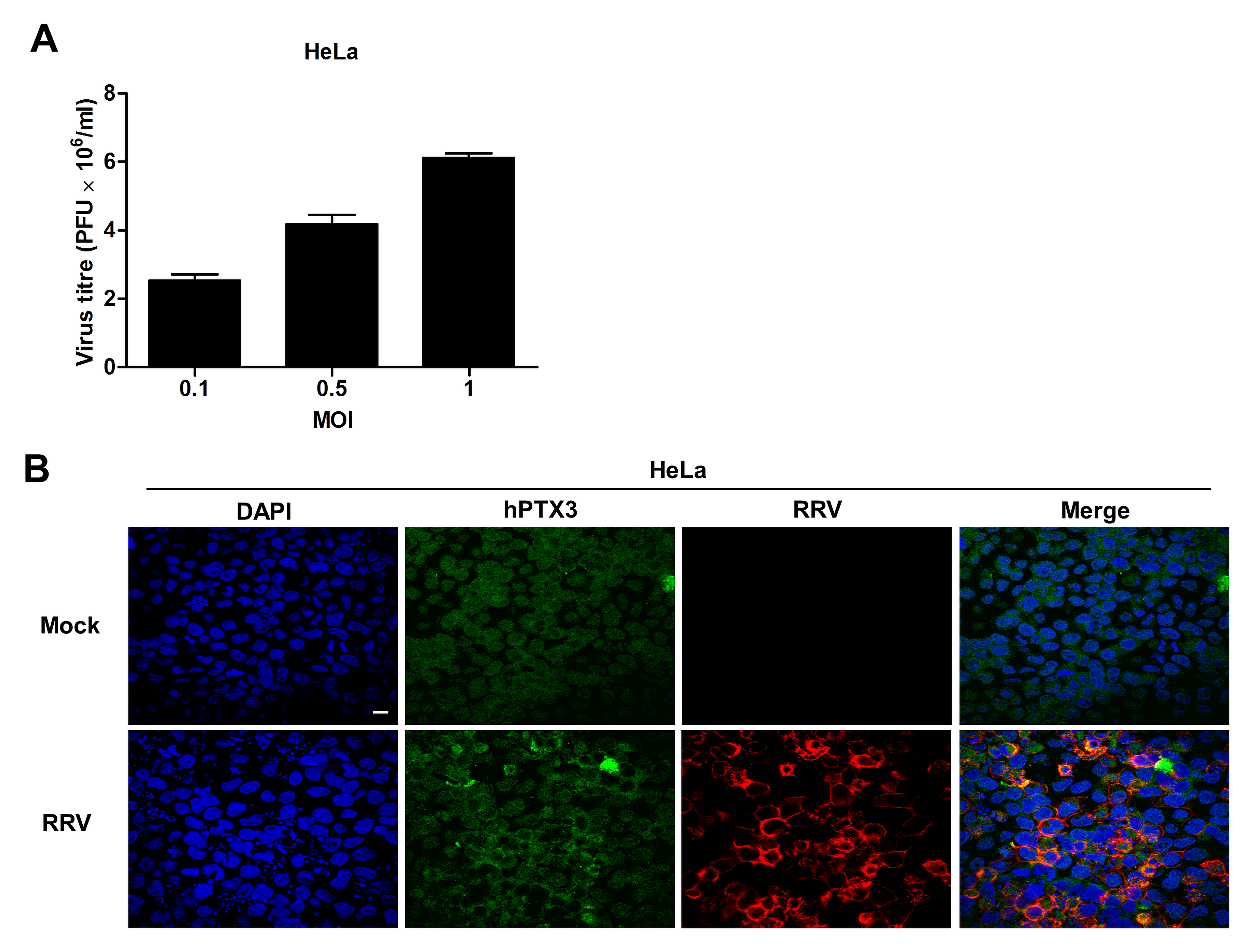

Supplement: S7 Fig — (A) Dose-dependent infection of HeLa cells with RRV were performed at MOI 0.1, 0.5 and 1 for 24 h. Supernatants were harvested and RRV titres determined by plaque assay on Vero cells. Data are presented as mean ± SEM. (B) HeLa cells were infected with RRV (MOI 1) and cells were harvested at 24 hpi, fixed and stained for PTX3 (green), RRV (red) and DAPI (blue). Images are representative of 2 independent experiments. Magnification, ×60. Scale bar, 10 μm. (TIF) [file ppat.1004649.s007.tif]
